# A cell-penetrating peptide blocks Toll-like receptor-mediated downstream signaling and ameliorates autoimmune and inflammatory diseases in mice

**DOI:** 10.1038/s12276-019-0244-0

**Published:** 2019-04-26

**Authors:** Hyuk-Kwon Kwon, Mahesh Chandra Patra, Hyeon-Jun Shin, Xiangai Gui, Asma Achek, Suresh Panneerselvam, Dong-Jin Kim, Suk-Jong Song, Riwon Hong, Kyoung Soo Kim, Yang Gyun Kim, Francis Y. Lee, Dae-Hyun Hahm, Sang Ho Lee, Sangdun Choi

**Affiliations:** 1 Department of Molecular Science and Technology, Ajou University, Suwon, 16499 Korea; 2 Division of Nephrology, Department of Internal Medicine, Kyung Hee University Hospital at Gangdong, Seoul, 05278 Korea; 3 Department of Science in Korean Medicine, College of Korean Medicine, Kyung Hee University, Seoul, 02447 Korea; 4 East-West Bone & Joint Research Institute, Kyung Hee University Hospital at Gangdong, Seoul, 05278 Korea; 5 Department of Orthopaedics and Rehabilitation, Yale School of Medicine, New Haven, CT 06510 USA; 6 Department of Physiology, School of Medicine, Kyung Hee University, Seoul, 02447 Korea; 7 Present Address: Department of Orthopaedics and Rehabilitation, Yale School of Medicine, New Haven, CT 06510 USA

**Keywords:** Toll-like receptors, Drug development

## Abstract

Toll-like receptors (TLRs) recognize pathogen/damage-associated molecular patterns and initiate inflammatory signaling cascades. Occasionally, overexpression of TLRs leads to the onset of numerous inflammatory diseases, necessitating the development of selective inhibitors to allow a protective yet balanced immune response. Here, we demonstrate that a novel peptide (TIP1) derived from Toll/interleukin-1 receptor (TIR) domain-containing adapter protein inhibited multiple TLR signaling pathways (MyD88-dependent and MyD88-independent) in murine and human cell lines. TIP1 also inhibited NLRP3-mediated IL-1β secretion, as we validated at both the protein and mRNA levels. Biophysical experiments confirmed that TIP1 specifically binds to the BB loop of the TLR4-TIR domain. Animal studies revealed that TIP1 inhibited the secretion of lipopolysaccharide (LPS)-induced proinflammatory cytokines in collagen-induced arthritis (CIA) and kaolin/carrageenan-induced arthritis (K/C) rodent models. TIP1 also rescued animals from sepsis and from LPS-induced kidney/liver damage. Importantly, TIP1 ameliorated the symptoms of rheumatoid arthritis in CIA and K/C rodent models, suggesting that TIP1 has therapeutic potential for the treatment of TLR-mediated autoimmune/inflammatory diseases.

## Inflammation: A decoy peptide treats arthritis and sepsis in mice

A newly discovered peptide drug that disrupts signaling through a critical immune pathway could offer a new treatment for inflammatory diseases. Sangdun Choi from Ajou University in Suwon, South Korea, and colleagues designed the drug to look similar to—and thus act as kind of decoy for—a protein that regulates signaling through Toll-like receptors (TLRs), proteins found on immune cells that initiate responses to pathogens and also play a role in autoimmunity. In mouse and human cells, the drug inhibited multiple TLR signaling pathways, including a pathway that normally activates a multiprotein complex implicated in a variety of inflammatory diseases. In mouse models of rheumatoid arthritis and sepsis, the drug limited the release of proinflammatory molecules in the blood and reduced disease symptoms.

## Introduction

Toll-like receptors (TLRs) are pattern-recognition receptors that play a fundamental role in the early detection of pathogen-associated molecular patterns and can sense damage-associated molecular pattern molecules from damaged cells^1^. These receptors have an extracellular ligand-binding domain which contains tandem leucine-rich repeats, a transmembrane (TM) domain, and an intracellular Toll/interleukin 1 receptor (TIR) domain. After ligand binding, TLRs dimerize and undergo structural changes required for the subsequent activation of downstream signaling. The specificity of TLR signaling lies not only in pathogen recognition but also in the specific engagement of downstream adaptor molecules. For instance, the recruitment of cytosolic TIR domain-containing adaptor protein (TIRAP; also known as MyD88 adapter-like [MAL]) induces subsequent activation of the myeloid differentiation primary response gene 88 (MyD88)-dependent signaling cascade. Initiation of the MyD88-dependent pathway leads to early-phase activation of nuclear factor kappa-light-chain enhancer of activated B cells (NF-κB) and secretion of proinflammatory cytokines. On the other hand, the recruitment of TIR domain-containing adapter-inducing interferon β (TRIF)-related adaptor molecule (TRAM) protein leads to triggering of the MyD88-independent signaling pathway and results in late-phase activation of NF-κB and secretion of type I interferon (IFN) via activation of interferon-regulatory factor (IRF)^2^. The arrangement of TLRs differs mainly according to the ligand recognized. For instance, TLR4 is expressed on the cell surface and is the only TLR with the ability to activate both MyD88-dependent and MyD88-independent signaling pathways upon binding to lipopolysaccharide (LPS)^3^. Furthermore, with the exception of TLR3, which initiates only the MyD88-independent signaling pathway, all TLRs activate their downstream signaling cascade via the MyD88-dependent pathway^4^.

The involvement of specific TLRs in the onset and/or progression of numerous diseases has been widely studied, and the pathophysiological roles of these receptors are well supported by a series of data from different studies^3^. In general, aberrant activation of TLRs leads to excessive secretion of proinflammatory cytokines^1,3,5,6^. On this topic, an increasing number of studies have emphasized the involvement of TLR4 and TLR3 in the development of arthritic conditions such as rheumatoid arthritis (RA) and osteoarthritis. The aberrant inflammatory response induced by activation of the aforementioned TLRs is suggested to be responsible for the excessive inflammation and massive tissue destruction observed in inflammatory diseases^6–8^. Moreover, TLR4 has been reported to be strongly associated with neurodegenerative diseases (specifically, Alzheimer’s disease and Parkinson’s disease) and atherosclerosis owing to its involvement in the formation of the NACHT, LRR, and PYD domains-containing protein 3 (NLRP3) inflammasome through the LPS-mediated activation of NF-κB, which subsequently leads to expression of NLRP3 and interleukin 1 beta (IL-1β)^9^. Thus, disruption of the inflammatory feedback loop resulting from excessive TLR responses should hinder inflammation and re-establish a physiologically appropriate immune response to pathogens. For these reasons, the development of TLR inhibitors, such as peptides, biochemical agents, or small molecules that can block the binding of ligands to the receptor or neutralize activation of the downstream adaptor molecules, holds promise as a powerful therapeutic strategy against TLR-related diseases^10,11^.

Despite the difficulties involved in the pursuit of drug development, it remains important to identify stable and effective molecules as future drug candidates. In the present study, we identified a novel TLR inhibitor (TLR-inhibitory peptide 1 [TIP1], derived from the TIR domain of TIRAP) that effectively inhibits cytokine secretion primarily elicited by the signaling pathways of TLR4 and TLR3 as well as TLR1/2 and TLR2/6 heterodimers, TLR7, TLR8, and TLR9. Cell-based assays showed that TIP1 successfully suppressed the production of proinflammatory cytokines and IFNs. Our findings were extended using in vivo models of sepsis and RA, confirming the anti-inflammatory properties of TIP1 and suggesting the protective role of the peptide.

## Materials and methods

### Cell culture and treatments

The HEK-Blue-hTLR4 and HEK-Blue IL-1R cell lines (InvivoGen, San Diego, CA, USA) were cultured in high-glucose Dulbecco’s modified Eagle’s medium (DMEM) containing 1% penicillin–streptomycin solution, 10% fetal bovine serum (FBS; Thermo Fisher Scientific, Inc., Waltham, MA, USA), and 0.2% Normocin (InvivoGen). RAW 264.7 cells (Korean Cell Line Bank, Seoul, Korea) were cultured in low-glucose DMEM containing 1% penicillin–streptomycin solution and 10% FBS (Thermo Fisher Scientific, Inc.). THP1 cells were cultured in RPMI 1640 medium containing 1% penicillin–streptomycin solution and 10% FBS (Thermo Fisher Scientific, Inc.) and were then differentiated into macrophages using 10 nM phorbol 12-myristate 13-acetate (PMA; Sigma-Aldrich, St. Louis, MO, USA) for 24 h. hPBMCs were purchased from PromoCell (Heidelberg, Germany) and cultured in RPMI 1640 containing 2.05 mM L-glutamine, 1% penicillin–streptomycin solution, and 10% FBS (Thermo Fisher Scientific, Inc.). hBMNCs were purchased from Lonza Inc. (Allendale, NJ, USA) and cultured in RPMI 1640 containing 2.05 mM L-glutamine, 1% penicillin–streptomycin solution, and 10% FBS (Thermo Fisher Scientific, Inc.). The experiments were performed on 6- to 7-week-old C57BL/6J mice (RAONBIO, Seoul, Korea) in a pathogen-free facility. After euthanasia of the mice by cervical dislocation, femurs and tibiae were collected with aseptic techniques. The ends were removed from the bones, and the marrow cavities were flushed out with DMEM by means of a sterile 26-gauge needle. The suspended cells were incubated at 37 °C in a 5% CO_2_ incubator for 1 h in complete medium (DMEM, 10% FBS, 100 U/ml penicillin, and 100 μg/ml streptomycin; Thermo Fisher Scientific, Inc.). Then, the marrow cells were cultured with macrophage colony-stimulating factor at 25 ng/ml (R&D Systems Inc., Minneapolis, MN, USA) for 3–4 days and harvested. Mouse bone marrow-derived macrophages (mBMDMs) were detached with trypsin-EDTA and plated in 60-mm dishes. The experiments were conducted after 24 h culture. All the cells were incubated in a humidified atmosphere containing 5% CO_2_ at 37 °C (Thermo Fisher Scientific, Inc.), and the media were changed after 16 h of incubation. PAM_3_CSK_4_, Poly(I:C), R848, and CpG-ODN were purchased from Thermo Fisher Scientific, Inc.; FSL-1 from InvivoGen; human IL-1β recombinant protein from eBioscience (San Diego, CA, USA); and LPS (*Escherichia coli* 0111:B4) and ATP from Sigma-Aldrich. All the peptides used in the experiments were synthesized by Peptron, Inc. (Daejeon, Korea).

### LPS-induced proinflammatory cytokine secretion in vivo

Eight-week-old C57BL/6 mice (20–25 g, *n* = 8) were acquired from Orient Bio, Inc. (Seoul, Korea). The mice were injected i.p. with TIP1 (10 nmol/[g body weight]) 1 h before being injected with LPS (5 μg/g). The control group was injected with the equivalent volume of PBS. Blood plasma samples were collected and stored at −80 °C until analysis for proinflammatory cytokines. For instance, the levels of secreted TNF-α, IL-12p40 (1:100 dilution), and IL-6 (1:100 dilution) cytokines were assessed using an ELISA kit (BioLegend, San Diego, CA, USA). The liver tissues analyzed in this study were homogenized by means of a Kontes Pellet Pestle Cordless Motor (Thermo Fisher Scientific, Inc.) containing the M-PER mammalian protein extraction reagent with a protease inhibitor cocktail (Thermo Fisher Scientific, Inc.) and then processed according to the manufacturer’s instructions. The protein expression of IL-6 and TNF-α was measured by western blotting; the method is described in detail in the section below. The levels of IL-6 and TNF-α protein expression were normalized to β-actin and analyzed using ImageJ software. All animal experiments were approved by the Institutional Animal Care and Use Committee (approval number: KHNMC AP 2016-006).

### LPS-induced sepsis in vivo

Eight-week-old C57BL/6 mice (20–25 g, *n* = 5) were purchased from Dae Han Bio Link Co., Ltd. (Seoul, Korea). The mice were injected i.p. with TIP1 (10 nmol/g) 1 h before the injection of LPS (5 μg/g). The control group was treated with an equivalent volume of phosphate-buffered saline (PBS). After 24 h, blood plasma samples were collected and stored at −80 °C until analysis of TNF-α and IL-6 (1:10 dilution) cytokine secretion. Cytokine secretion was assessed using an ELISA kit (eBioscience). Moreover, the levels of blood urea nitrogen (BUN), creatinine (Cr), aspartate aminotransferase (AST), and alanine aminotransferase (ALT) in the plasma samples were measured with a VETTEST-8008 system (IDEXX, Ludwigsburg, Germany). The mice were perfused with PBS for at least 5 min to remove the blood from the kidneys, after which the kidneys were weighed on an electronic balance (ER-180A, A & D Company, Tokyo, Japan). Kidney tissue slices were obtained by cutting along the sagittal plane. The tissues were fixed in a 10% formalin solution overnight, embedded in paraffin wax, and cut into 4-μm-thick slices on a microtome. Apoptotic cells in the kidneys were quantified by staining them with a TUNEL Staining Kit (Merck Millipore, Billerica, MA, USA) and then examining them by confocal microscopy (LSM-700, Carl Zeiss Microscopy GmbH, Munich, Germany) using the Zen 2009 software package. All animal experiments were approved by the Institutional Animal Care and Use Committee (approval number: KHNMC AP 2016-006).

### The survival rate under the influence of LPS in the in vivo model

Eight-week-old BALB/c male mice (20–25 g, *n* = 10 or 6) were injected i.p. with TIP1 (10 nmol/g) 1 h before the administration of LPS (5 or 10 μg/g). The control group was injected with an equivalent volume of PBS. The day of injection was referred to as day 0, and the survival rates were recorded for up to 5 days postinjection. All the animal experiments were approved by the Institutional Animal Care and Use Committee (approval number: KHNMC AP 2016-006 and KHNMC AP 2017-007).

### RA in a model of CIA

All animal care and experimental procedures were conducted in accordance with the National Institute of Health *Guide for the Care and Use of Laboratory Animals* and were approved by the Animal Care and Use Committee of Kyung Hee University [Permit number: KHUASP(SE)-15-115]. Male DBA/1J mice weighing 20–23 g (6–7 weeks old) were purchased from Central Lab Animal Inc. (Seoul, Korea). The mice were housed in a limited-access rodent facility at 22–24 °C with up to four animals per polycarbonate cage under a 12:12-h light/dark cycle with free access to pelleted food and water. CIA was implemented according to the protocol previously described^12^. Briefly, the mice were immunized at the base of the tail with a mixture of 100 μg of chicken type II collagen (CII, Sigma-Aldrich) and an equal volume of complete Freund’s adjuvant (Sigma-Aldrich); this time point was designated as day 0. The mice were then given a booster (second) injection of the mixture on day 14. All mice were subdivided randomly into seven experimental groups (*n* = 8): (1) untreated normal group (NOR); (2) vehicle-treated arthritis group (CIA, *n* = 8); TIP1-treated arthritis groups, namely, (3) a group treated with 2.5 nmol/g TIP1 and designated CIA-TIP1 (2.5 nmol/g), (4) a group treated with 5 nmol/g TIP1 and designated CIA-TIP1 (5 nmol/g), and (5) a group treated with 10 nmol/g TIP1 and designated CIA-TIP1 (10 nmol/g); (6) an arthritis group treated with 5 μg/g prednisolone and designated CIA-prednisolone (5 μg/g), and (7) a group treated with 10 nmol/g TIP1 in the postarthritis phase and designated PAP CIA-TIP1 (10 nmol/g). TIP1 (2.5, 5, or 10 nmol/g) or prednisolone dissolved in saline was i.p. injected once a day starting on day 15 (the day after the second collagen injection). To evaluate arthritis progression in the CIA mice, body weight, paw volume, squeaking score, and the arthritic index were measured. The mouse body weights were measured using a digital balance (Mettler-Toledo Inc., Columbus, OH, USA). Joint pain in the hindlimb was evaluated by scoring squeaking during forced flexion and extension of the hindlimb ankle joint 10 times every 5 s. A score of 0 (no vocalization) or 1 (vocalization) was assigned to each hindlimb for every flexion and extension procedure. Total numbers of vocalizations detected by the observer were then calculated as the squeaking score. Paw volume was measured by the volume displacement of an electrolyte solution using a water-displacement plethysmometer (Ugo-Basile Biological Research Apparatus Co., Comerio-Varese, Italy) as described elsewhere^13,14^. The hind paw was immersed to the level of the hairy skin, and the volumes were read on a digital display. Paw volume was expressed as a relative value compared with that on day 0, which was defined as 1.0 (100%). The arthritis index was assessed by grading the apparent arthritis severity of all joints of the limbs on a four-point scale per mouse: 0 = no erythema or swelling of any joint in one limb, 1 = erythema or swelling of at least one joint per limb, 2 = erythema or swelling of fewer than three joints per limb, 3 = erythema or swelling of all joints in one limb, and 4 = ankylosis and deformity of all joints in one limb. The maximal score was 16 for each mouse. The behavioral tests were performed twice a week on each animal. The mice were euthanized on day 45 for joint tissue sampling. Immunohistochemical staining was conducted to evaluate the degree of immune-cell infiltration into the affected joints. Mouse knee joints were dissected, fixed for 3 days in 10% formalin, dehydrated through a graded ethanol series, cleared in xylene, and processed for embedding in paraffin wax by routine protocols. Coronal sections (8 µm thick) through the knee joint were prepared on a manual rotary microtome (Finesse 325; Thermo Shandon Inc., Pittsburgh, PA, USA). The slices were stained with H&E for routine histological evaluation. The inflammation severity was evaluated on a scale of 0–5 by three pathologists who were blinded to the group assignments. The scale was defined as follows: 0 = no inflammation, 1 = mild inflammation, 2 = mild-to-moderate inflammation, 3 = moderate inflammation, 4 = moderate-to-severe inflammation, and 5 = severe inflammation. The samples of bone were fixed with a 10% formalin solution to prepare them for micro-CT analysis. Bone specimens were scanned in the knee joint by means of an in vivo micro-CT (NFR Polaris-G90; NanoFocus Ray Co., JeonJu, Korea). The following settings were selected: X-ray voltage of 55 kV, X-ray current of 105 μA, X-ray spot size of 8 μm, and exposure time of 80 ms for each of the 180° rotational steps. Reconstruction of the 3D images of the knee joints, 2D images of the trabecular bone in the sagittal section at the top of the tibia, and the cortical bone in the horizontal section in the middle of tibia was performed in Data viewer, CTVox, and CTAn software packages (SkyScan, Kontich, Belgium) on 500 slices. The bone mineral density of each reconstructed knee joint was measured at 1 mm from the top of the tibia after setting the region of interest.

### The model of K/C-induced knee monoarthritis

Adult male Sprague–Dawley rats weighing 180–200 g (6 weeks old) were purchased from Central Lab Animal Inc. (Seoul, Korea). All animal experiments were approved by the Institutional Animal Care and Use Committee (approval number: KHNMC AP 2016-009). The rats were housed in a limited-access rodent facility at 22–24 °C, with up to five rats per polycarbonate cage, under a 12:12-h light/dark cycle, with free access to pelleted food and water. A K/C-induced monoarthritis model was generated according to the protocol previously described^13,14^. Briefly, acute arthritis was unilaterally induced by a single injection (into the left knee joint) of 5% carrageenan + 5% kaolin resuspended in 100 mL of pyrogen-free sterile saline. The rats were distributed at random into several groups: untreated (naïve) rats (i.e., normal group, *n* = 5), vehicle-treated arthritic rats after K/C injection (K/C group, *n* = 5), 2.5 nmol/g TIP1-treated arthritic rats after K/C injection (K/C-TIP1 2.5 group, *n* = 5), 10 nmol/g TIP1-treated arthritic rats after K/C injection (K/C-TIP1 10 group, *n* = 5), and 10 nmol/g TIP1-treated rats before K/C injection (Pre K/C-TIP1 10 group, *n* = 5). TIP1 and vehicle were i.p. administered once a day for 5 days starting on day 1, 2 h before every behavioral assessment. Only in the Pre K/C-TIP1 10 group was the TIP1 injection started on day 0, 1 h before K/C injection. Arthritis severity was evaluated by means of relative swelling and weight-bearing deficit of the affected hindlimb. Hindlimb swelling was measured by the volume displacement of an electrolyte solution using a water-displacement plethysmometer (Ugo-Basil Biological Research Apparatus Co., Comerio-Varese, Italy) as described elsewhere^13^. A hindlimb was immersed to the level above the knee joint, and the volume change was read on a digital display. The hindlimb’s volume was expressed as a relative value (%) toward that on day 0, which was defined as 100%. Arthritic pain was assessed by measuring weight-bearing loading of two hindlimbs of arthritic rats, that is, a weight distribution ratio (WDR). WDR was measured using an incapacitance meter (UGO-BASILE Biological Research Apparatus Co.) and calculated as follows: %WDR = 100 × (weight borne by affected limb ÷ total weight borne by both limbs). The load-bearing difference between the two hindlimbs was quantified by two mechanotransducers separately placed below two hind legs: one was normal, and the other was the affected limb. The load borne by each hind leg was estimated as a 5-s average, and the mean bearing force was calculated from four separate experiments. The WDR of the normal rats was 50:50, indicating that 50% of the total weight was carried by each hindlimb. As arthritic pain progressed, the weight-bearing balance was disrupted, resulting in a decrease in the WDR of the arthritic hindlimb. All behavioral tests were conducted blinded.

### Cell viability

This parameter was measured using a colorimetric 1-(4,5-dimethylthiazol-2-yl)-3,5-diphenylformazan (MTT) solution (Sigma-Aldrich), and the assay was carried out as described previously^15^. HEK-Blue-hTLR4 cells were seeded at a density of 5 × 10^4^/well, RAW 264.7 cells at a density of 2 × 10^5^/well, and hBMNCs at a density of 4 × 10^4^/well. All cells were grown overnight in 96-well plates (BD Biosciences, San Jose, CA, USA).

### SEAP activity

HEK-Blue-hTLR4 cells were seeded at a density of 2 × 10^5^ cells/well and grown overnight in 24-well plates (BD Biosciences). The next day, the supernatant was removed, and the medium was replaced with fresh medium. Aliquots of supernatants (200 µl) from the treated cells were transferred to microcentrifuge tubes and heated for 10 min at 65 °C on a heating block (FINEPCR Co., Seoul, Korea). The supernatants were then transferred into new 96-well plates (BD Biosciences), and SEAP production was quantified using the HEK-Blue detection kit (InvivoGen). HEK-Blue IL-1R cells were seeded at a density of 2 × 10^5^ cells/well and grown in 24-well plates (BD Biosciences) overnight, and SEAP production was measured by means of QUANTI-Blue (InvivoGen). Absorbance was measured on a microplate reader (spectrophotometry system; Molecular Devices Inc., Silicon Valley, CA, USA) or a Cytation 5 Cell Imaging Multi-Mode Reader (Bio-Tek Instruments, Winooski, VT, USA) at 620 nm.

### Western blotting

Total protein extraction was performed using the M-PER mammalian protein extraction reagent (Thermo Fisher Scientific, Inc.). The concentration of proteins was measured with the bicinchoninic acid (BCA) assay kit (Sigma-Aldrich). Western blot analysis, including gel electrophoresis and transfer, was conducted by means of the Mini-PROTEAN Tetra Cell and Mini Trans-Blot electrophoretic transfer cell system (Bio-Rad Laboratories, Hercules, CA, USA)^15^. The membranes were immunoblotted with specific primary antibodies (1:500–1000) against p-p65, p65, p-JNK, JNK, p-IRF3, IRF3, p-TBK1, TBK1, p-ERK, ERK, p-p38, p38, Iκ-Bα, and human IL-1β (Cell Signaling Technology Inc., Danvers, MA, USA); ATF3, COX2, caspase 1, and β-actin (Santa Cruz Biotechnology Inc., Dallas, TX, USA); iNOS (BD Biosciences); IL-6 and mouse IL-1β (R&D Systems Inc.); TNF-α (Thermo Fisher Scientific, Inc.); or NLRP3 (Adipogen, San Diego, CA, USA) with gentle shaking at 4 °C overnight. Next, the membranes were rigorously washed with PBST and incubated with a peroxidase-conjugated anti-mouse or anti-rabbit immunoglobulin G antibody (1:1000) for 2 h. The proteins were detected by means of a SuperSignal West Pico ECL solution (Thermo Fisher Scientific, Inc.) and visualized on a Fuji LAS-3000 system (Fujifilm, Tokyo, Japan) or ChemiDoc Touch Imaging System (Bio-Rad Laboratories).

### Confocal microscopy

RAW 264.7 and THP1 cells were seeded at a density of 2 × 10^5^ cells/well in 24-well plates (BD Biosciences) containing coverslips and grown overnight. After RAW 264.7 cells were treated with LPS for 30 min, the cells were fixed in a 3.7% formaldehyde solution (Sigma-Aldrich Co. LLC, St. Louis, MO, USA) for 15 min and permeabilized with a 0.2% Triton X-100 solution (AMRESCO, Solon, OH, USA) for 15 min. After that, the cells were washed three times with PBS and blocked by means of a 2% BSA solution (Thermo Fisher Scientific, Inc.). Next, the cells were incubated with antibodies against p-p65 (1:1000; Santa Cruz Biotechnology Inc.) for 2 h and washed with PBS three times. The cells were incubated with an Alexa Fluor 488-conjugated secondary antibody (Invitrogen, Carlsbad, CA, USA) for 1 h and washed with PBS three times. Incubation with a Hoechst 33258 solution (5 µM; Sigma-Aldrich Co., LLC) for 15 min was used to stain nuclei. After the LPS-primed THP1 cells were treated with ATP for 1 h, they were fixed in a 3.7% formaldehyde solution (Sigma-Aldrich Co., LLC) for 15 min and permeabilized with a 0.2% Triton X-100 solution (AMRESCO) for 15 min. Then, the cells were washed three times with PBS and blocked by means of a 2% BSA solution (Thermo Fisher Scientific, Inc.). Next, the cells were incubated with antibodies against TOM20 (1:1000; Santa Cruz Biotechnology Inc.) and NLRP3 (1:1000; Adipogen) for 2 h and washed with PBS three times. The cells were incubated with Alexa Fluor 488- and 546-conjugated secondary antibodies (Invitrogen) for 1 h and washed with PBS three times. Incubation with a Hoechst 33258 solution (5 µM; Sigma-Aldrich Co., LLC) for 15 min was conducted to stain nuclei. RAW 264.7 cells were treated with FITC-conjugated TIP 1 (25 µM; Peptron, Inc., Daejeon, Korea) for 1 h and then treated with LPS for 30 min. The cells were fixed in a 3.7% formaldehyde solution (Sigma-Aldrich Co., LLC) for 15 min and permeabilized with a 0.2% Triton X-100 solution (AMRESCO) for 15 min. Next, the cells were washed three times with PBS and blocked by means of a 2% BSA solution (Thermo Fisher Scientific, Inc.). The cells were incubated with antibodies against TLR4 and MyD88 (1:1000; Santa Cruz Biotechnology, Inc.) for 2 h and washed with PBS three times. The cells were then incubated with Alexa Fluor 408- and 546-conjugated secondary antibodies (Invitrogen) for 1 h and washed with PBS three times. Fluorescence intensities were measured by confocal microscopy (LSM-700; Carl Zeiss Microscopy GmbH), and images were analyzed in Zen 2009 software.

### TNF-α, IL-6, IFN-β, and IL-1β

RAW 264.7, THP1, and mBMDM cells were seeded at a density of 2 × 10^5^ cells/well in 96-well plates (BD Biosciences) or at 5 × 10^5^ cells/well in 24-well plates (BD Biosciences) and were grown overnight. After 24 h of treatment, IFN-β secretion was measured by means of the LEGEND MAX Mouse IFN-β precoated ELISA Kit (BioLegend), IL-6 secretion was assessed by a Mouse IL-6 ELISA MAX Deluxe (BioLegend) or Mouse IL-6 Platinum ELISA (eBioscience), TNF-α production was assessed using the Mouse TNF alpha ELISA Ready-SET-Go! kit (eBioscience), and the IL-1β cytokine level was measured with the Mouse or Human IL-1β ELISA Ready-SET-Go! kit (eBioscience). hBMNCs were seeded at a density of 4 × 10^4^/well in 96-well plates (BD Biosciences) and then grown overnight. After 24 h of treatment, the secretion levels of IL-6 and TNF-α were assessed using Human IL-6 ELISA MAX Deluxe (BioLegend) and Human TNF alpha ELISA MAX Deluxe (BioLegend), respectively. The plates were then analyzed on a microplate spectrophotometer system (Molecular Devices) at the appropriate wavelength.

### Intracellular NO and ROS

RAW 264.7 cells were seeded at 10^6^ in 6-cm dishes (SPL Life Sciences, Pochun, Korea) and grown overnight. After treatment, intracellular NO and ROS were quantified by means of DAF-FM and DCF-DA dyes (Thermo Fisher Scientific, Inc.) as described elsewhere^16^. The fluorescence intensity of the cells was analyzed on a FACSAria III instrument with Diva software (BD Biosciences).

### NO secretion

RAW 264.7 and mBMDM cells were seeded at a density of 2 × 10^5^/well and grown overnight in 96-well plates (BD Biosciences). Secretion of NO was measured using the Nitric Oxide (NO) Detection Kit (iNtRON Biotechnology Inc., Seongnam, Korea)^15^. The absorbance in plates was read on the microplate spectrophotometry system (Molecular Devices) at 550 nm.

### SPR

The SPR experiments were conducted using a ProteOn XPR36 instrument (Bio-Rad Laboratories, Inc.) with a ProteOn NLC sensor chip. PBST served as running buffer, and 0.85% phosphoric acid or PBST was used for regeneration. Synthetic peptides encoding the BB loop (710-RDFIPGVAIAA-720) of the TIR domain were immobilized by NeutrAvidin bound to the GLC polymer layer onto surfaces of an NLC sensor chip as described in the protocol. Different concentrations of TIP1 (12.5, 25, or 50 µM) were run into the chip to check their binding with the immobilized peptides. Running buffer was injected into an empty channel as a reference. The dissociation was monitored for 5 min, and the chip was regenerated for the second round. ProteOn Manager software (version 2.0) was employed to analyze the data. The binding curves were processed for the start injection alignment and baseline. A reference-subtracted sensorgram was fitted globally to the curves describing a homogeneous 1:1 model. Data from various doses of TIP1 were grouped together to fit the kinetic rate constants (*k*_a_ and *k*_d_). The equilibrium dissociation constant, *K*_D_, was calculated according to the equation *K*_D_ = *k*_d_/*k*_a_.

### Construction of three-dimensional (3D) model of TIP1

The 3D structure of the TIP1 peptide was constructed using Discovery Studio (DS) Visualizer (Dassault Systèmes BIOVIA, San Diego, CA, USA) based on the secondary structure predicted by the PSIPRED server^17^. Since TIP1 is a cell-translocating peptide, it was energy minimized and simulated over a dipalmitoylphosphatidylcholine (DPPC) bilayer to obtain a biophysically relevant tertiary geometry in the presence of membrane phospholipids. Molecular dynamics (MD) simulation of the peptide–membrane system was performed in GROMACS 5.0.7^18^ by placing the peptide 10 Å above the bilayer surface. The initial and final structural models of TIP1 after MD simulation are shown in Supplementary Fig. 5.

### MD simulation of TIP1 over a DPPC bilayer

TIP1 was conjugated with a cell-penetrating peptide to facilitate membrane insertion and translocation. Therefore, MD simulation of modeled TIP1 was carried out over a pre-equilibrated DPPC bilayer consisting of 288 phospholipids. The peptide and membrane were represented by GROMOS96 54A7 and Berger lipid force fields, respectively. The peptide was oriented and placed 10 Å above the upper leaflet of the bilayer using VMD software^19^. Appropriate numbers of water molecules were added to fully solvate the peptide–membrane system and physiological strength (0.15 M) of counterions was added for charge neutralization by replacing random water molecules. Energy minimization was conducted using the steepest descent algorithm followed by position restraint simulations through NVT and NPT ensembles for temperature and pressure equilibration, respectively. Then, a 100 ns production run was carried out without backbone restraints using the NPT ensemble. Short-range (van der Waals) interactions were calculated with a distance cutoff of 12 Å via the Verlet scheme, and long-range (electrostatic) interactions were handled by applying the particle mesh Ewald algorithm. The simulation was performed using a periodic boundary condition. Temperature and pressure couplings were achieved using Nose-Hoover at 300 K and the Parrinello-Rahman method at 1 bar, respectively. All bonds were constrained using the LINC algorithm, and coordinates were saved at every 2 ps. Trajectory data analysis was conducted using VMD and Grace [http://plasma-gate.weizmann.ac.il/Grace/]. The initial and final snapshots of the TIP1-membrane simulation system are shown in Supplementary Fig. 6.

### Protein–protein docking

The interaction between TIP1 and TLR4-TIR domains was studied by a protein–protein docking method. An optimized low-energy model of TIP1 (see Supplementary Figs. 5 and 6) was docked to the TIR domain of a recently published TLR4 model^20^ using the ZDOCK server^21^. Due to the size limitation in ZDOCK, a truncated TLR4 including a fraction of the membrane containing complete TM and TIR domains was defined as the receptor. Docking was performed without specifying any binding or blocking regions on either the receptor or ligand. The best docking solution was superimposed on the full-length TLR4 structure to obtain the complete TLR4-TIP1 docked complex. The complex was then energy minimized in a membrane-aqueous environment before analysis of the intermolecular interactions.

### Statistical analysis

All data analyses involved the *t* test in the SigmaPlot software, version 12.0 (Systat Software Inc., San Jose, CA, USA) or GraphPad Prism 5 (GraphPad Software, Inc., CA, USA).

## Results

### Screening of potential TLR4 inhibitors derived from the TIR domain of TIRAP

**Fig. 1:**
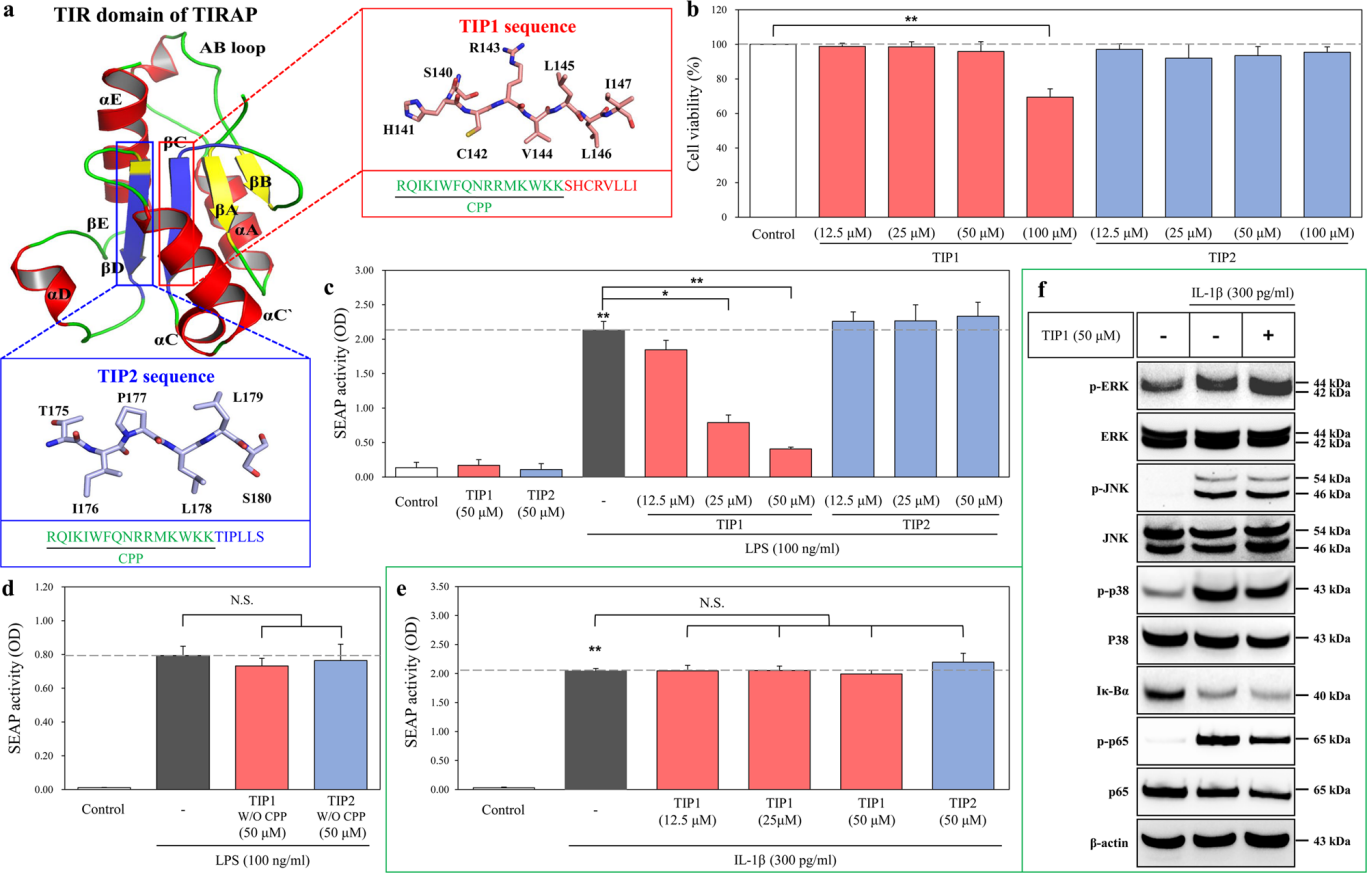
Design of the TLR decoy peptides derived from the TIR domain of TIRAP. **a** TLR-inhibitory peptides (TIP) 1 and 2 were designed using the sheet structures of βC (red box) and βD (blue box), respectively, and then conjugated with cell-penetrating peptide (CPP; green color). **b** HEK-Blue hTLR4 cells were treated with various concentrations of TIP1 (12.5, 25, 50, or 100 µM) or TIP2 (12.5, 25, 50, or 100 µM) for 24 h. Cell viability was measured on a microplate reader by the MTS assay. **c** The activation of NF-κB was measured by a SEAP activity assay after the treatment of HEK-Blue hTLR4 cells with the different indicated concentrations of peptides for 1 h in the presence or absence of LPS for 24 h. **d** The activation of NF-κB was measured by the SEAP activity assay after the treatment of HEK-Blue hTLR4 cells with the TIP1 or -2 peptide not conjugated with CPP: TIP1 or TIP2 without (W/O) CPP. **e** NF-κB activity was measured by a SEAP activity assay upon the treatment of HEK-Blue IL-1R cells with different concentrations of peptides for 1 h before the treatment with IL-1β (300 pg/ml) for the next 24 h. **f** HEK-Blue IL-1R cells were treated with TIP1 (50 µM) for 1 h in the presence or absence of IL-1β (300 pg/ml) for 30 min. The amounts of p-ERK, ERK, p-JNK, JNK, p-p38, p38, Iκ-Bα, p-p65, and p65 were measured by western blotting in the total protein extract. β-Actin served as a loading control. The data shown represent at least three independent experiments (*n* ≥ 3), and bars represent means ± SEM (*P < 0.05, **P < 0.01). N.S.: not significant

The TLR4-mediated response to LPS leads to a direct interaction between the TIR domains of TIRAP and MyD88, resulting in the subsequent activation of the MyD88-dependent downstream cascade. On the other hand, LPS-induced activation of TLR4 can cause the interaction between the TIR domains of TRAM and TRIF, which thereafter initiates MyD88-independent downstream signaling^22^. Multiple peptides were designed from the TIR domain of TIRAP to possibly target the TIR domain of TLR4. Because peptides with α-helical or β-sheet structures are more stable than linear peptides, we designed peptides from β-sheet structures via a structural analysis approach considering stability and solubility factors; the designed molecules were named “TIP” (Fig. 1a). TIP1 (sequence SHCRVLLI) and TIP2 (sequence TIPLLS) were conjugated in tandem to a cell-penetrating peptide (CPP) of the *Drosophila* antennapedia homeodomain sequence (RQIKIWFQNRRMKWKK)^23^ at their N terminus to facilitate their intracellular uptake and ensure their efficient delivery to the target protein (Fig. 1a). Analysis of cytotoxicity of TIP was performed by the MTS (3-(4,5-dimethylthiazol-2-yl)-5-(3-carboxymethoxyphenyl)-2-(4-sulfophenyl)-2H-tetrazolium) assay on HEK-Blue hTLR4 cells in a dose-dependent manner in the range of 12.5–100 μM. Although TIP2 did not show any significant cytotoxicity at any of the concentrations tested, TIP1 had cytotoxic effects at the concentration of 100 μM but did not exert any toxic effect at concentrations ranging from 12.5 to 50 μM (Fig. 1b). Therefore, based on these findings, further experiments were conducted at concentrations ranging from 12.5 to 50 μM. Moreover, to study the effect of TIP1 on the TLR4-induced signaling pathway after LPS stimulation, we proceeded to measure NF-κB activity by a secreted alkaline phosphatase (SEAP) activity assay, which was performed on HEK-Blue hTLR4 cells. Our data revealed that TIP1 hampered LPS-induced SEAP activity in a dose-dependent manner, whereas TIP2 did not hinder LPS-induced NF-κB activity (Fig. 1c). The inhibitory effects of the peptides (TIP1 and TIP2) in the absence of CPP were evaluated by measuring the SEAP activity in HEK-Blue hTLR4 cells. As expected, neither peptide significantly inhibited NF-κB activity when compared with the activity observed after LPS stimulation (Fig. 1d). It is known that TLR and the interleukin-1 receptor (IL-1R) superfamily share a conserved cytoplasmic domain and that the binding of IL-1β to IL-1R induces activation of NF-κB and mitogen-activated protein kinases (MAPKs), including extracellular signal-regulated kinase (ERK), c-Jun N-terminal kinase (JNK), and p38 mitogen-activated protein kinase (p38), through the interaction between the TIR domain and MyD88^8,24^. To evaluate the specific binding of TIP1 to TLRs, we measured the NF-κB activity and protein expression of NF-κB and MAPKs by the SEAP activity assay and western blotting in HEK-Blue IL-1R cells. The results revealed that the treatment of cells with IL-1β induced NF-κB activity in the SEAP assay; however, treatment with either TIP1 or TIP2 did not exert any significant inhibitory effects (Fig. 1e). Similarly, the IL-1β-mediated activation of NF-κB [degradation of inhibitor of NF-κB α (Iκ-Bα) and phosphorylation of p65 (p-p65)] and the phosphorylation of MAPKs [ERK (p-ERK), JNK (p-JNK), and p38 (p-p38)] were not hindered by TIP1 (Fig. 1f). Taken together, these data suggested that TIP1 could be a promising TLR4 inhibitor, which once translocated into the intracellular compartment, interferes with downstream adaptor molecules and blocks the activation of the TLR4-mediated signaling cascade, independently from the IL-1R pathway.

### The inhibitory effects of TIP1 on the TLR signaling pathway

**Fig. 2:**
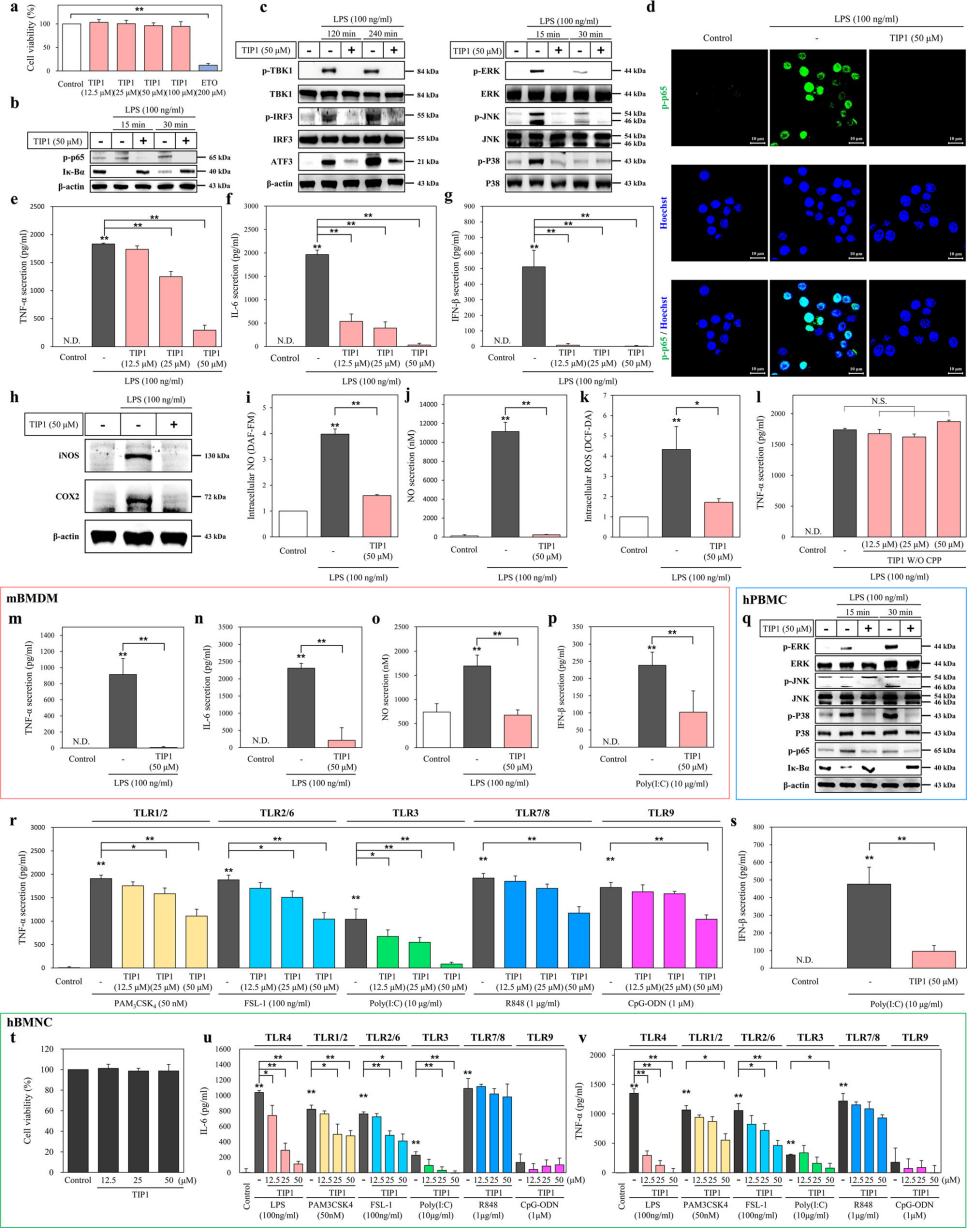
Inhibitory effects of TIP1 on TLR signaling pathways. **a** Cell viability was measured by the MTT assay upon the treatment of RAW 264.7 cells with various concentrations of TIP1 (12.5, 25, or 50 µM) for 24 h. Etoposide (ETO) served as a positive control because of its high cytotoxicity. **b**, **c** The cells were treated with TIP1, followed by treatment with LPS as indicated. The amounts of p-p65, Iκ-Bα, p-TBK1, TBK1, p-IRF3, IRF3, ATF3, p-ERK, ERK, p-JNK, JNK, p-p38, and p38 were measured by western blotting in the total protein extract. β-Actin served as a loading control. **d** The amount of p-p65 was measured by immunofluorescent staining and confocal microscopy. Hoechst 33258 was used for nucleus staining (scale bar represents 10 μm). **e**–**g** Cells were treated with TIP1 (12.5, 25, or 50 µM) in the presence or absence of LPS for 24 h. The secretion levels of TNF-α, IL-6, and IFN-β were measured by ELISAs. **h** The expression levels of iNOS and COX2 were measured by western blotting, and β-actin served as a loading control. **i**, **j** The production levels of intracellular and cytosolic NO were measured by DAF-FM staining and an NO secretion kit, respectively. **k** The production of intracellular ROS was measured by DCF-DA staining. **l** The secretion of TNF-α was measured by an ELISA after the treatment of RAW 264.7 cells with different concentrations of TIP1 not conjugated with CPP (12.5, 25, and 50 µM; TIP1 without CPP). **m**–**o** mBMDM cells were treated with TIP1 for 1 h in the presence or absence of LPS for 24 h. The secretion levels of (**m**) TNF-α and (**n**) IL-6 were measured by an ELISA, and the production of NO (**o**) was evaluated using an NO secretion kit. **p** mBMDM cells were treated with TIP1 for 1 h, followed by treatment with Poly(I:C) for 24 h. The secretion of IFN-β was measured by an ELISA. **q** hPBMCs were treated with TIP1 in the presence or absence of LPS. The amounts of p-ERK, ERK, p-JNK, JNK, p-p38, p38, p-p65, and Iκ-Bα were measured by western blotting. β-Actin was used as a loading control. (**r**) The secretion of TNF-α was measured by an ELISA during treatment with PAM_3_CSK_4_ (affecting TLR1/2), FSL-1 (affecting TLR2/6), Poly(I:C) (affecting TLR3), R848 (affecting TLR7–TLR8), or CpG-ODN (affecting TLR9) at diverse concentrations. **s** The secretion of IFN-β was measured by an ELISA during Poly(I:C) (TLR3) treatment. **t** Cell viability was assessed by the MTT assay after the treatment of hBMNCs with various concentrations of TIP1 (12.5, 25, or 50 µM) for 24 h. **u**, **v** The secretion levels of IL-6 and TNF-α were measured by ELISAs after treatment of the hBMNCs with different concentrations of TIP1 for 1 h and LPS (affecting TLR4), PAM_3_CSK_4_ (affecting TLR1/2), FSL-1 (affecting TLR2/6), Poly(I:C) (affecting TLR3), R848 (affecting TLR7 and/or TLR8), or CpG-ODN (affecting TLR9) for 24 h. The data shown represent at least three independent experiments (*n* ≥ 3), and bars denote mean ± SEM (*P < 0.05, **P < 0.01). N.D.: not detected. N.S.: not significant

In the MyD88-dependent signaling pathway, the early-phase activation of NF-κB results in the secretion of proinflammatory cytokines, such as tumor necrosis factor α (TNF-α) and interleukin 6 (IL-6)^25^. Nevertheless, the MyD88-independent signaling pathway leads to activation of TRAF family member-associated NF-κB activator (TANK)-binding kinase 1 (TBK1)-mediated interferon-regulatory factors (IRFs) 3 and 7 and to the late-phase secretion of type I IFNs such as IFN-α and -β^25^. In addition, it is known that stimulation of TLRs by their cognate ligands induces activation of MAPKs: ERK, JNK, and p38; this effect subsequently stimulates transcription factor activator protein 1 (AP-1) and leads to the production of proinflammatory cytokines and type I IFNs^26^. Similarly, TLR4 stimulation leads to the induction of cyclooxygenase 2 (COX2) and inducible nitric oxide synthase (iNOS) as well as the production of nitric oxide (NO) in mouse macrophagelike (RAW 264.7) cells^5,27^. Some studies have shown that the expression of activating transcription factor 3 (ATF3) increases after TLR4 stimulation by LPS in RAW 264.7 cells^25,28^. In primary macrophage cells, LPS has been reported to successfully induce mitochondrial and intracellular generation of reactive oxygen species (ROS)^29,30^. Based on these findings, we examined the modulating effect of TIP1 on the TLR4 signaling pathway after LPS stimulation in RAW 264.7 cells. Cell viability analysis indicated that TIP1 did not have any cytotoxic effects. The chemotherapeutic drug etoposide served as a positive control in the cell viability experiment (Fig. 2a). The inhibitory effects of TIP1 on the TLR4-mediated pathway were then evaluated at the highest concentration, 50 μM. Western blot data indicated that TIP1 completely suppressed LPS-induced MyD88-dependent and -independent signaling pathways by inhibiting degradation of Iκ-Bα and phosphorylation of p65 (p-p65), TBK1 (p-TBK1), and IRF3 (p-IRF3; Fig. 2b, c). As expected, TIP1 also attenuated the upregulation of ATF3 by LPS (Fig. 2c). Similarly, TIP1 hampered the expression of MAPKs by lowering p-ERK, p-JNK, and p-p38 levels in LPS-treated RAW 264.7 cells (Fig. 2c). Confocal microscopy analysis confirmed that TIP1 attenuated the LPS-induced p-p65 upregulation in the nucleus (Fig. 2d). Ultimately, the LPS-induced production of proinflammatory cytokines (IL-6 and TNF-α) and type I IFN (IFN-β) was significantly hindered by TIP1 in a dose-dependent manner (Fig. 2e–g). TIP1 also successfully attenuated LPS-induced iNOS and COX2 expression (Fig. 2h). Similarly, the intracellular production of NO and ROS as well as the extracellular secretion of NO were inhibited by TIP1 (Fig. 2i–k). Taken together, these findings revealed that TIP1 significantly hindered LPS-induced TLR4-mediated activation of MyD88-dependent and -independent signaling pathways in RAW 264.7 cells.

Next, we evaluated the inhibitory effect of TIP1 on primary human peripheral blood mononuclear cells (hPBMCs). As in the abovementioned data on RAW 264.7 cells, TIP1 completely suppressed LPS-induced p65 phosphorylation and expression of MAPKs and COX2 and inhibited Iκ-Bα degradation (Fig. 2q, Supplementary Fig. 1a). In primary mouse bone marrow-derived macrophage cells (mBMDMs), TIP1 clearly suppressed LPS-induced secretion of TNF-α and IL-6 and reduced extracellular generation of NO (Fig. 2m–o). Furthermore, to assess the effects of TIP1 on other TLR family members, we performed an enzyme-linked immunosorbent assay (ELISA) and evaluated the secretion levels of TNF-α after treatment of RAW 264.7 cells with TIP1 and a ligand for TLR1/2 (PAM_3_CSK_4_), TLR2/6 (FSL-1), TLR3 [polyinosine-polycytidylic acid; Poly(I:C)], TLR7–TLR8 (resiquimod; R848), or TLR9 (CpG oligodeoxynucleotide; CpG-ODN). Of note, TIP1 significantly diminished TLR3-mediated TNF-α secretion in a dose-dependent manner (92% reduction at 50 μM) and slightly hindered TLR1/2-, TLR2/6-, TLR7-, TLR8-, and TLR9-mediated TNF-α secretion in RAW 264.7 cells (Fig. 2r). Similarly, TIP1 strongly reduced the Poly(I:C)-induced TLR3-mediated secretion of IFN-β in both RAW 264.7 cells and mBMDM cells (Fig. 2p–s). We also tested incubation of primary human bone marrow mononuclear cells (hBMNCs) with TIP1 and a cognate ligand of a targeted TLR, e.g., LPS, PAM_3_CSK_4_, FSL-1, Poly(I:C), R848, or CpG-ODN. Cell viability analysis indicated the absence of any cytotoxic effect of TIP1 on hBMNCs (Fig. 2t). TIP1 significantly decreased the TLR4- and TLR3-mediated IL-6 and TNF-α secretion levels in a dose-dependent manner and suppressed the TLR1/2- and TLR2/6-mediated secretion of both IL-6 and TNF-α (Fig. 2u, v). On the other hand, removal of CPP sequences from TIP1 and TIP2 resulted in the loss of the inhibitory effect of both peptides on TNF-α secretion (Fig. 2l, Supplementary Fig. 1c).

### TIP1 diminishes the secretion of IL-1β by inhibiting the expression of NLRP3

**Fig. 3:**
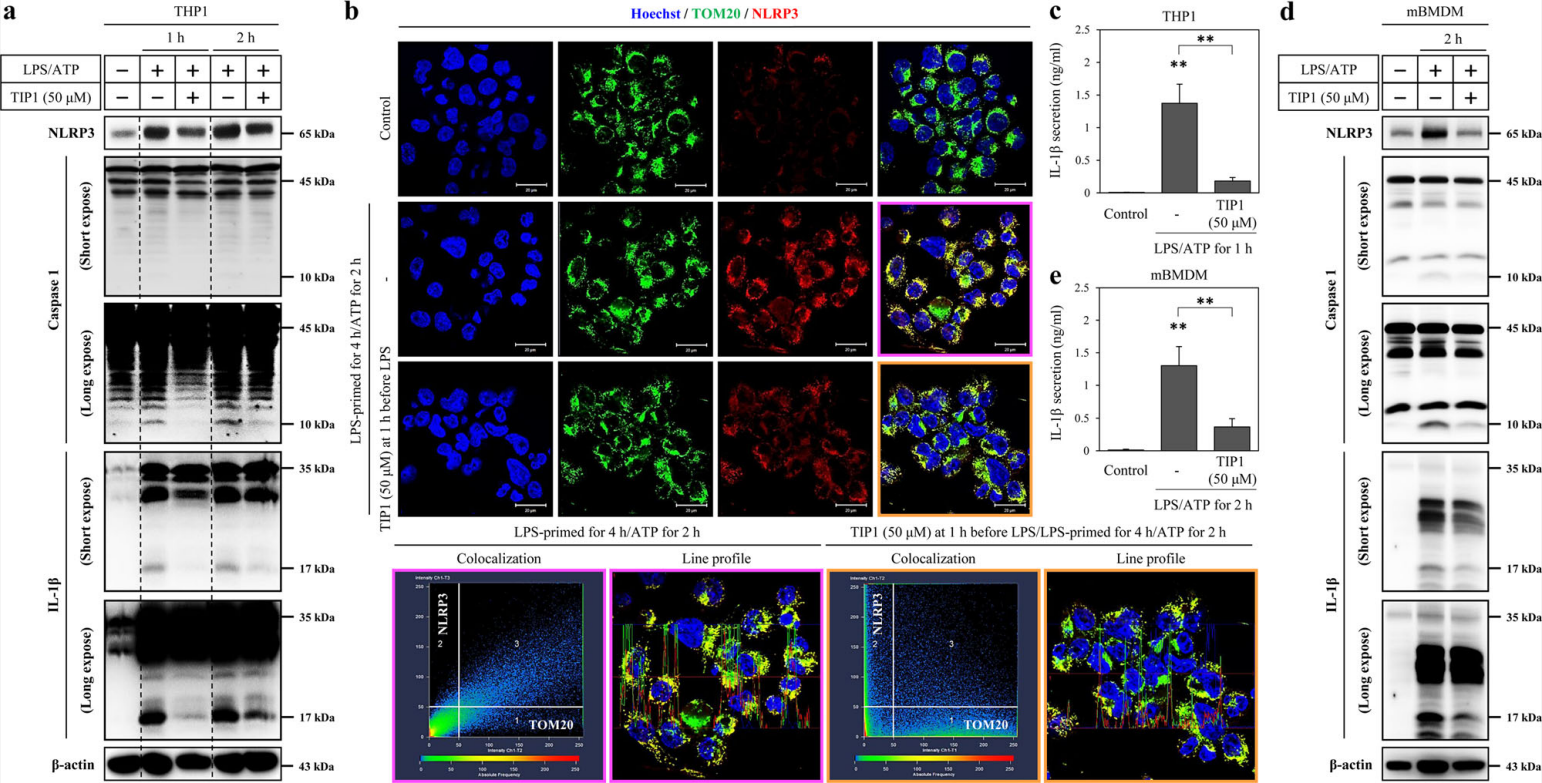
Inhibitory effects of TIP1 on NLRP3-mediated responses. The antagonistic effects of TIP1 were evaluated in LPS-primed (1 μg/ml for 4 h) THP1 cells treated with ATP (5 mM). When TIP1 treatment was required, TIP1 was added at 1 h before LPS treatment. **a** The expression levels of NLRP3, procaspase 1 (45 kDa), active caspase 1 (10 kDa), pro-IL-1β (35 kDa), and mature IL-1β (17 kDa) were measured by western blotting at 1 and 2 h. β-Actin served as a loading control. **b** The expression of TOM20 and NLRP3 was measured by immunofluorescent staining and confocal microscopy. Hoechst was used for nucleus staining (scale bar represents 20 μm). The scatterplot shows the correlation of TOM20 and NLRP3 channels, and the line profile image shows fluorescence intensity for TOM20, NLRP3, and Hoechst. **c** The secretion of IL-1β in THP1 cells was evaluated by ELISA. **d** LPS-primed (1 μg/ml for 4 h) mBMDM cells were treated with ATP (5 mM) for 2 h. The expression levels of NLRP3, procaspase 1 (45 kDa), active caspase 1 (10 kDa), pro-IL-1β (35 kDa), and mature IL-1β (15 kDa) were measured by western blotting. β-Actin was used as a loading control. **e** The secretion of IL-1β by mBMDM cells was measured by an ELISA. The data shown represent at least three independent experiments (*n* ≥ 3), and bars denote the mean ± SEM (*P < 0.05, **P < 0.01)

Triggering of the TLR4 signaling pathway by LPS mainly activates NF-κB, which subsequently drives the expression of NOD-like receptor (NLR), NACHT, LRR, and PYD domains-containing protein 3 (NLRP3), and interleukin 1β (IL-1β) in macrophage cells^9^. Under these conditions, adenosine triphosphate (ATP) and potassium efflux agents decrease the intracellular potassium level and the production of mature IL-1β by the NLRP3 inflammasome, which is a complex of NLRP3, an apoptosis-associated specklike protein containing a caspase activation and recruitment domain (ASC), and procaspase 1^9^. Therefore, due to the close association of TLR4 activation with NLRP3 inflammasome formation, we treated macrophages derived from human monocytic cells (THP1 cells) with TIP1 for 1 h, followed by treatment of LPS-primed cells with LPS or with ATP for 4 h. We next evaluated the effect of TIP1 on the expression of NLRP3, procaspase 1 (45 kDa), active caspase 1 (10 kDa), pro-IL-1β (35 kDa), and matured IL-1β (17 kDa). LPS-induced expression of NLRP3 and pro-IL-1β (35 kDa) was attenuated by treatment with TIP1 for 4 h, but procaspase 1 (45 kDa) expression did not manifest any significant inhibition (Supplementary Fig. 1b). In LPS-primed THP1 cells, treatment with ATP increased the expression levels of NLRP3, active caspase 1 (10 kDa), pro-IL-1β (35 kDa), and mature IL-1β (17 kDa; Fig. 3a). In comparison with LPS-primed cells treated with ATP, TIP1 remarkably suppressed the intracellular expression of NLRP3, active caspase 1 (10 kDa), and matured IL-1β (17 kDa) and eventually blocked IL-1β secretion (Fig. 3a–c). Similarly, in mBMDM cells treated with LPS and with ATP, TIP1 notably repressed the intracellular expression of NLRP3, active caspase 1 (10 kDa), and matured IL-1β (17 kDa) and IL-1β secretion (Fig. 3d, e). Some studies have shown that NLRP3 interacts with mitochondria, thus activating the canonical and noncanonical NLRP3 inflammasome and contributes to the maturation of IL-1β and pyroptotic or apoptotic cell death^15,31^. According to these data, we measured the expression of NLRP3 and colocalization of NLRP3 with mitochondrial import receptor subunit translocase of outer membrane 20 (TOM20) by immunofluorescent staining upon treatment of the LPS-primed THP1 cells with ATP for 2 h. As in the western blot results, the data suggested that the treatment of cells with ATP increased the expression of NLRP3, and it colocalized with TOM20: this process was inhibited by treatment with TIP1 (Fig. 3b). Therefore, TIP1 notably blocked IL-1β maturation mediated by the NLRP3 inflammasome through inhibition of LPS-induced NLRP3 and IL-1β expression levels.

### TIP1 binds to the BB loop of the TIR domain

**Fig. 4:**
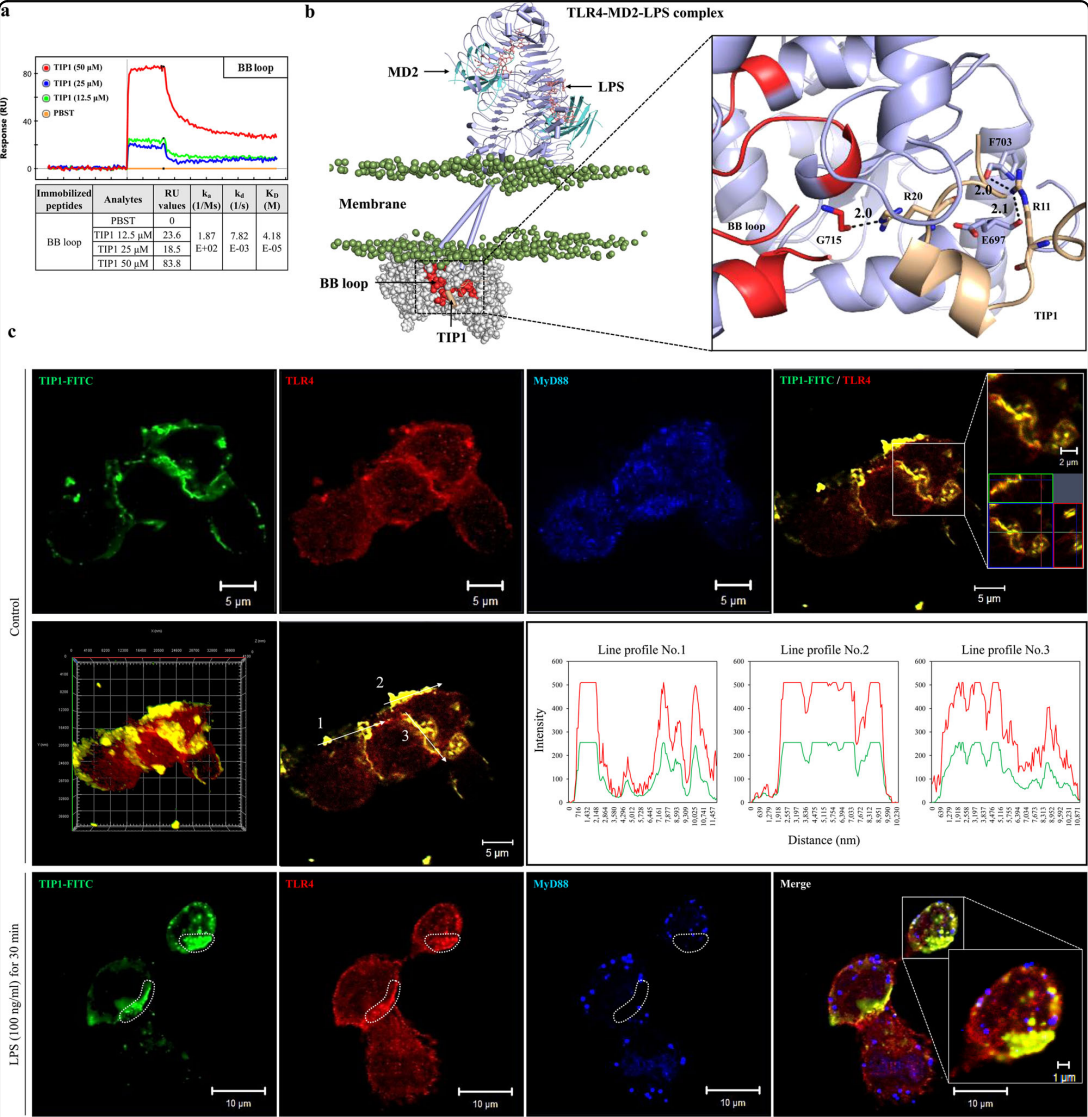
Characterization of the antagonistic effects of TIP1. **a** The potential binding of TIP1 to the BB loop of the TIR domain, as confirmed by SPR analysis. The peptide sequence of the BB loop (710-RDFIPGVAIAA-720) was conjugated with biotin in tandem to facilitate coating of the sensor chip with the peptides. **b** An illustration of TIP1 binding to the BB loop region of the TLR4-TIR domain. The BB loop is colored red and TIP1 light yellow. The TLR4 extracellular and TM domains are shown as the cylindrical-cartoon model, and the TIR domain is shown as the space-filling model. LPS is colored orange, and phosphate atoms are shown as mauve beads. The hydrogen bond (H-bond)-forming residues are shown as stick models. Digits indicate the distance between H-bond forming atoms in the angstrom unit. **c** THP1 cells were treated with FITC-conjugated TIP1 for 1 h before treatment with LPS (100 ng/ml) for 30 min. The expression levels of TLR4 and MyD88 and the localization of TIP1-FITC were determined by immunofluorescent staining and confocal microscopy. Orthogonal projections, 3D image reconstruction, and fluorescence intensity profiles of confocal images show localization of TIP1-FITC and TLR4

Surface plasmon resonance (SPR) is a powerful technique used for high-throughput screening and is considered a potent tool for drug discovery. SPR can provide detailed information on peptide–receptor binding kinetics or small-molecule–protein or protein–protein interactions^32^. The potential binding interface and affinity between TIP1 and the TLR4-TIR domain were confirmed by SPR analysis, which supported the hypothesis that binding of TIP1 to the BB loop of the TLR4–TIR domain blocks the downstream signaling cascade via MyD88 or TRIF adaptors. The BB loop sequence (710-RDFIPGVAIAA-720) was conjugated in tandem with biotin and applied to a sensor chip surface. The association rate constant (*k*_a_), dissociation rate constant (*k*_d_), and equilibrium dissociation constant (*K*_D_) of TIP1 were measured in a dose-dependent manner (Fig. 4a). TIP1 (50 μM) interacted with the BB loop with an RU value of 83.8. In addition, the *K*_D_ value (which is calculated as *k*_d_/*k*_a_) was 40.18 μM, suggesting that TIP1 has a fairly higher binding affinity for the BB loop. On the other hand, the DD loop sequence, which was taken as a negative control, showed inconsistent binding with the different peptide sequences of TIP1 (data not shown). To confirm the binding of TIP1 to TLR4, we conjugated fluorescein isothiocyanate (FITC) to the N terminus of TIP1. THP1 cells were treated with TIP1-FITC for 1 h before the treatment of the cells with LPS for 30 min. Later, the fluorescence intensities of TIP1-FITC, TLR4, and MyD88 were measured by immunofluorescent staining and confocal microscopy. In the absence of LPS, TLR4 was expressed on the plasma membrane, whereas MyD88 spread into the intracellular compartment. TIP1-FITC was also located in the plasma membrane and almost perfectly colocalized with TLR4 but not with MyD88 (Fig. 4c). Nonetheless, the treatment of cells with LPS-induced internalization of both TLR4 and TIP1-FITC into the intracellular compartment by endocytosis, and the two were colocalized. On the other hand, MyD88 did not spread into the intracellular compartment and was found aggregated on the plasma membrane. While TIP1-FITC-TLR4 showed strong binding, MyD88 yielded weak binding to TIP1-FITC (the dotted area in Fig. 4c). Therefore, these data suggested that the inhibitory effect of TIP1 on TLR4-mediated downstream signaling was due to its binding to the BB loop regions of the TIR domain, and this action in turn blocked the interaction of TLR4 with TIRAP and MyD88 as well as with TRAM and TRIF, thereby antagonizing the initiation of downstream signaling. In addition, we performed protein–protein docking to identify the key residues that might be involved in the interaction between TIP1 and the TIR domain of TLR4. In congruence with the SPR data, analysis of a representative docked conformation over the BB loop revealed that TIP1 forms three high-affinity hydrogen bonds (H-bonds) with the TLR4-TIR domain. Specifically, R20 forms an H-bond with G715 of the BB loop. In addition, R11 from the CPP interacts with F703 and E697 of the AB loop (Fig. 4b). This indicates that TIP1 could directly target the BB loop together with its neighboring regions and prevent the association of TLR4 with the adaptors.

### In vivo analysis of the therapeutic effect of TIP1 on inflammation and sepsis

**Fig. 5:**
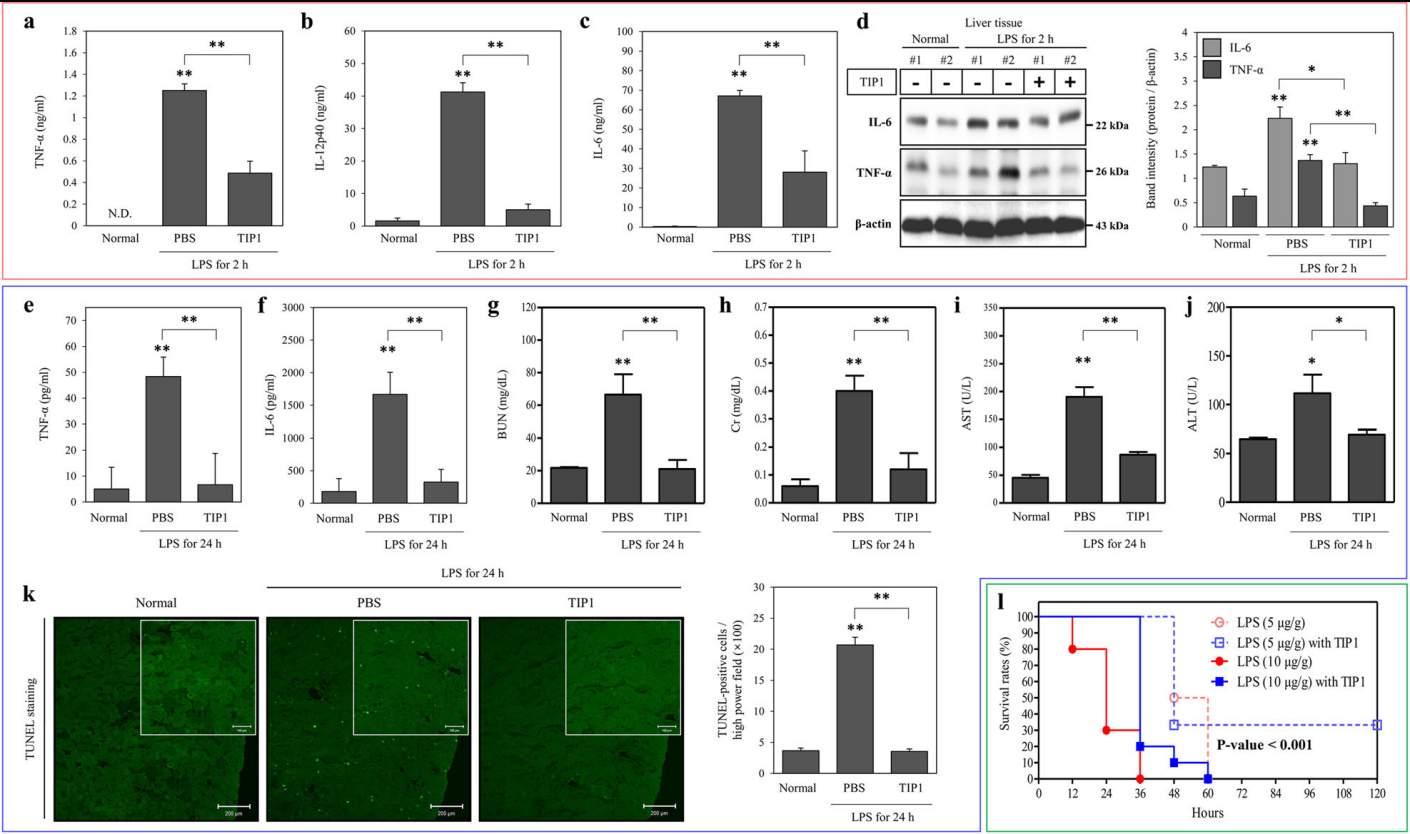
TIP1 alleviates LPS-induced inflammation in in vivo sepsis models. **a**–**d** C57BL/6J male mice (*n* = 8) were i.p. injected with PBS or 10 nmol/g of TIP1 1 h before i.p. administration of LPS (5 µg/g). Measurements of (**a**) TNF-α, (**b**) IL-12p40, and (**c**) IL-6 secretion were conducted on plasma samples after 2 h. **d** The protein levels of TNF-α and IL-6 were measured by western blotting in liver tissue. β-Actin was utilized as a loading control. Histograms represent the band intensities of TNF-α and IL-6 normalized to β-actin. **e–l** C57BL/6J male mice (*n* = 5) were i.p. injected with PBS or 10 nmol/g TIP1 1 h before i.p. administration of LPS (5 µg/g). **e**, **f** Evaluation of TNF-α or IL-6 levels was performed by an ELISA on plasma samples after 24 h. **g**–**j** Levels of biological markers of renal dysfunction [blood urea nitrogen (BUN) and creatinine (Cr)] and markers of liver dysfunction [aspartate aminotransferase (AST) and alanine aminotransferase (ALT)] were measured in plasma samples. **k** Representative photographs of apoptotic cells in the kidney after TUNEL staining (scale bars represent 200 or 100 μm) and the quantitatively measured scores of TUNEL-positive cells in the histogram. **l** BALB/c male mice were i.p. injected with PBS or 10 nmol/g TIP1 1 h before i.p. administration of LPS (5 or 10 µg/g), and the survival rates were recorded for 5 days postinjection. The data shown represent at least three independent experiments (*n* ≥ 3), and bars denote the mean ± SEM (*P < 0.05, **P < 0.01). N.D.: not detected

To test the effectiveness of TIP1 in vivo, C57BL/6J mice were injected intraperitoneally (i.p.) with TIP1 and later injected with LPS at a dose of 5 µg/g. TIP1 was injected at a dose of 10 nmol/g 1 h prior to the LPS challenge. Serum samples were collected from the treated mice after 2 h and then used to evaluate the secretion levels of TNF-α, IL-12 p40 (IL-12p40), and IL-6 by ELISAs. Our data indicated a significant increase in the secretion levels of TNF-α, IL-12p40, and IL-6 after treatment with LPS. As expected, pretreatment with TIP1 suppressed LPS-induced secretion of TNF-α, IL-12p40, and IL-6 in the mouse model (Fig. 5a–c).

The mouse model of LPS-induced sepsis is a popular in vivo model because of its ability to reproduce the pathogenesis of the human disease^33^. Moreover, the relevance of this animal model has been validated by several clinical features observed during different stages of the disease, such as kidney and liver failure and other inflammation-related signs^34,35^. Herein, we next analyzed the anti-inflammatory effect of TIP1 on the animal model of sepsis. C57BL/6J mice were i.p. injected with TIP1 (10 nmol/g) for 1 h followed by i.p. injection of LPS (5 µg/g) for 24 h. Proinflammatory cytokine secretion was assessed by an ELISA performed on plasma samples collected from the treated mice. As expected, the injection of LPS into mice increased the levels of TNF-α and IL-6. However, treatment of these mice with TIP1 significantly diminished the secretion of these cytokines (Fig. 5e, f). To further assess the inhibitory effects of TIP1 on multiple-organ failure, we measured the biological markers of kidney and liver functions in tissue samples. First, the levels of BUN and Cr in the serum samples were measured as indicators of kidney dysfunction. BUN and Cr levels notably increased upon LPS injection in the mice and then were clearly decreased by pretreatment with TIP1 (Fig. 5g, h). Furthermore, we performed a terminal deoxynucleotidyl transferase dUTP nick end labeling (TUNEL) assay to evaluate the effect of TIP1 on DNA fragmentation resulting from apoptotic signaling cascades. Our results suggested that treatment with LPS led to a significant increase in the number of TUNEL-positive kidney cells and therefore an increase in the number of apoptotic kidney cells. In contrast, pretreatment with TIP1 significantly downregulated TUNEL-positive apoptotic cells (Fig. 5k). After that, the protein expression of TNF-α and IL-6 in liver tissue was evaluated to further evaluate the negative effect of TIP1 on liver function. The protein levels of TNF-α and IL-6 remarkably increased after the treatment of mice with LPS. As expected, TIP1 injection resulted in a significant reduction in the protein expression of TNF-α and IL-6 (Fig. 5d). In general, AST and ALT are commonly used in biological assays of liver function. As expected, AST and ALT activities in serum samples collected from LPS-injected mice were high, indicating liver damage. Serum samples from TIP1-treated mice, however, showed a significant decrease in ALT and AST activities, indicating that the treatment of mice with TIP1 alleviated the liver failure caused by LPS-induced inflammation (Fig. 5i, j). Finally, to confirm the therapeutic effect of TIP1 on the animal model of sepsis, BALB/c male mice were injected i.p. with TIP1 (10 nmol/g) for 1 h followed by i.p. injection of LPS (5 and 10 µg/g). The mice were closely monitored for 5 days to evaluate their survival rate. The mouse model of mild sepsis (LPS injected at a concentration of 5 µg/g) showed a 100% decrease in the survival rate within 3 days, but treatment with TIP1 preserved a 30% survival rate and maintained it for 5 days (the mice survived up to 7 days; data not shown). Furthermore, the model of severe sepsis (LPS injected at a concentration of 10 µg/g) showed a 70% decrease in 1-day survival (3/10), and all the mice died within the next 2 days. In contrast, in the TIP1-treated group, all the mice survived during the first day of treatment, followed by the death of most of the mice (9/10) within the following 2 days (Fig. 5l). Our data suggest that TIP1 improved the survival rate of mice by alleviating inflammation at the initial stage. Thus, TIP1 suppressed the development of sepsis by suppressing inflammation and alleviating liver and kidney failure in mice, implying potential protection from sepsis because of the prevention of the death of mice under the influence of overwhelming inflammation.

### In vivo evaluation of the therapeutic effects of TIP1 in rodent models of collagen-induced arthritis (CIA) and kaolin/carrageenan-induced arthritis (K/C)

**Fig. 6:**
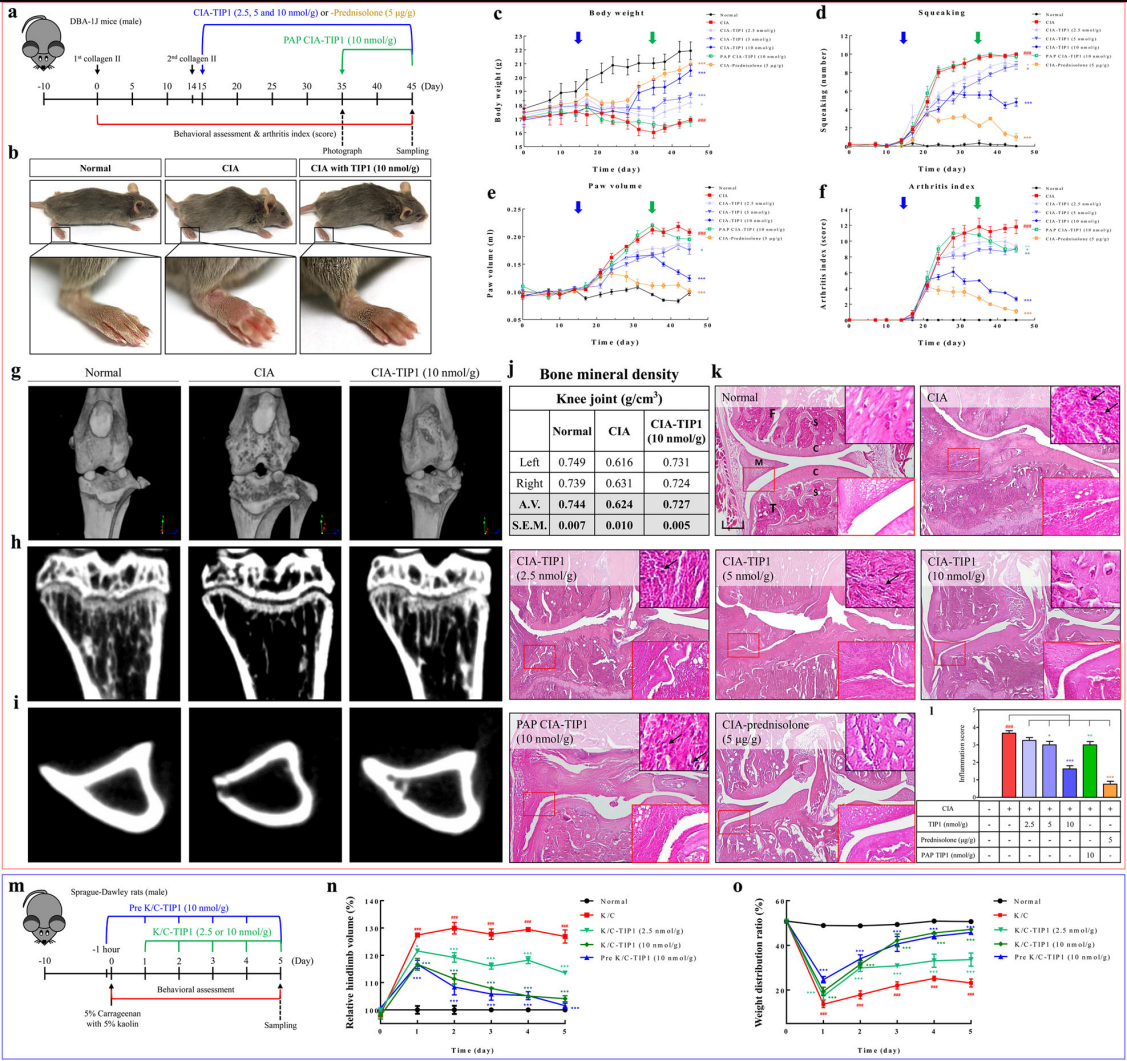
Protective effects of TIP1 against CIA and K/C in rodent models. **a** A summary of the experimental validation of the inhibitory effect of TIP1 in the RA model. DAB-1J male mice (*n* = 9) were first subcutaneously (s.c.) injected with type II collagen and then s.c. injected with type II collagen after 14 days. Two types of experiments were designed. First, mice were daily i.p. injected with one of three concentrations of TIP1 (2.5, 5, or 10 nmol/g) or, as a positive control, prednisolone (5 mg/kg), for 30 days. Second, starting on day 25, the mice were daily i.p. injected with TIP1 (10 nmol/g) for 10 days to test the postarthritis phase (PAP). (**b**) On day 45, photographs showing the overall representative shapes of the paws were taken, and then magnified features of right hind paws were evaluated. **c**–**f** During the experiments, the mice were analyzed for (**c**) body weight, (**d**) squeaking, (**e**) paw volume, and (**f**) RA index. Blue and green arrows indicate two time points of TIP1 treatment: starting the injection on day 15 (blue arrow) before the development of arthritic inflammation or on day 35 (green arrow) after full development of arthritis, respectively. Numerical data are presented as the mean ± SEM: ^#^*P* < 0.05, ^##^*P* < 0.01, and ^###^*P* < 0.001 CIA vs. Normal; *P < 0.05, **P < 0.01, and ***P < 0.001 TIP1 vs. CIA. **g** Representative 3D images of the knee joints were measured using micro-CT. Representative 2D images of (**h**) trabecular bone corresponding to the sagittal section at the top of tibia and (**i**) cortical bone corresponding to the horizontal section in the middle of tibia were measured by micro-CT. **j** The bone mineral densities (BMD) of the right or left knee joints were measured by micro-CT. **k** Representative sections of knee joints from normal, CIA, CIA with TIP1 (2.5, 5, or 10 nmol/g), and CIA with prednisolone (5 μg/g) were evaluated by hematoxylin and eosin (H&E) staining to show synovial hyperplasia (original magnification × 40). The black scale bar indicates 200 μm (× 40 magnification). Insets in the upper right corners show inflammatory cell infiltration. Small red squares in H&E-stained images are magnified in the bottom right insets (× 200). C: cartilage, S: subchondral bone, F: femur, T: tibia, M: meniscus. **l** Histograms present quantification of synovial hyperplasia scores. **m**–**o** To demonstrate the in vivo therapeutic effects of TIP1, the antiarthritic efficacy of TIP1 was tested in a rat model of acute arthritis induced by kaolin/carrageenan (K/C) injection. **m** Monoarthritis was induced in one knee by a single injection of 5% carrageenan + 5% kaolin (dissolved in 100 μl of saline) into the intra-articular space of the right knee joint on day 0. Each dose of TIP1 was i.p. administered daily for 5 days starting on day 1, 2 h before every measurement of arthritic symptoms such as pain and edema. **n** Arthritic edema was measured with the digital Plethysmometer and was expressed as a relative change in arthritic-paw volume toward the unaffected paw. **o** Arthritic pain was quantified with the incapacitance meter by determining weight-bearing forces of both hindlimbs (weight distribution ratio; WDR). The data shown represent at least three independent experiments (*n* ≥ 3), and bars represent means ± SEM; ^###^*P* < 0.001: CIA vs. normal, and *P < 0.05, **P < 0.001, ***P < 0.001: TIP1 vs. CIA

Numerous studies on the TLR4-dependent mechanisms of arthritic diseases such as RA and osteoarthritis have been conducted to identify the mechanisms underlying the inflammation response observed during these diseases and thereby to facilitate the discovery of new therapeutic agents. In the present study, the protective effect of TIP1 against LPS-induced sepsis in the mouse model points to TIP1’s potential as a therapeutic agent for a wide range of inflammatory diseases. Next, we investigated the effect of TIP1 on chronic inflammatory diseases such as RA in a CIA mouse model. To create the experimental model of CIA, DAB-1J male mice were first injected with type II collagen subcutaneously in the base of the tail and then injected with type II collagen after 14 days. We designed two types of TIP1 treatment schedules: (1) after the second injection of collagen (on day 15), we started daily injection of TIP1 (2.5, 5, or 10 nmol/g) or prednisolone (5 mg/kg) as a positive control for 30 days, (2) after full development of arthritis (on day 35), we started TIP1 injection (10 nmol/g) for 10 days (postarthritis phase, PAP) (Fig. 6a).

To examine RA severity, we tested several parameters, such as body weight, squeaking, paw volume, and the arthritis index. Between days 20 and 30, arthritic symptoms were clearly visible in all limbs, according to the images showing the swelling of the knee, ankle, and foot. Nevertheless, the induction of RA was significantly attenuated in TIP1-injected mice compared with CIA mice (Fig. 6b, Supplementary Fig. 2). During the entire period of arthritis development, the body weights of CIA mice did not change (average: 16.9 g), whereas those of normal mice increased (average: 21.9 g). The loss of body weight was not observed in TIP1 (10 nmol/g)- and prednisolone-injected mice (average 20.5 and 21.0 g, respectively), whose body weights were almost the same as those of normal mice (Fig. 6c). The measurements of squeaking and paw volume in the normal, CIA, and TIP1-injected mice showed that these two parameters were highly increased in the CIA mice compared with normal mice but not in the TIP1 (10 nmol/g)- or prednisolone-injected mice (Fig. 6d, e). The arthritis index also increased in the CIA mice, but the injection of TIP1 (10 nmol/g) or prednisolone significantly decreased the index score (Fig. 6f). In the case of 30-day injection of TIP1 (10 nmol/g), the development of arthritis, indicated by paw volume and the arthritis index, was significantly inhibited when compared with the CIA mice, but body weight and squeaking number did not significantly change. After that, to confirm the extent of bone erosion, we captured three-dimensional (3D) images of knee joints and 2D images of trabecular and cortical bones from the normal, CIA, and TIP1 (10 nmol/g)-injected mice during in vivo micro-computed tomography (micro-CT) scanning. Severe erosion of bone and cartilage in the knee joint, loss of bone microarchitecture, depression of cortical thickness, and reduction of bone mineral density were observed in the CIA mice compared with the normal mice. Similarly, the results of behavioral analysis revealed that TIP1 reliably inhibited the bone destruction observed in the CIA mice (Fig. 6g–j, Supplementary Fig. 3). Immune-cell infiltration, thicker synovial tissues, synovial hyperplasia, pannus formation, and joint space narrowing were clearly visible on histological examination of joint tissue sections stained with hematoxylin and eosin (H&E) in CIA mice. In contrast, the 10 nmol/g TIP1 treatment effectively prevented these histopathological aberrations in a dose-dependent manner. These histological observations were similar to the data from micro-CT images (Fig. 6k, l).

To evaluate the in vivo preventive and therapeutic effects of TIP1, we additionally tested the antiarthritic efficacy of the peptide in a rat model of acute arthritis induced by K/C injection^13,14^. We designed two types of treatment schedules (Fig. 6m). First, on day 0, rats were injected with TIP1 (10 nmol/g) 1 h before the injection of K/C to examine the preventive effect; for the next 5 days, daily injections of TIP1 (10 nmol/g) were carried out (group Pre K/C-TIP1; total six injections of TIP1). Second, on day 0, a K/C injection was administered, and for the next 5 days, daily injections of TIP1 (2.5 or 10 nmol/g) were carried out to study the therapeutic effect of the peptide (group K/C-TIP1; five injections of TIP1). One day after the injection of K/C, the hindlimb volume and the weight distribution ratio (WDR) reached their maximal levels, and this pattern persisted for 5 days in the K/C group (i.e., the “K/C one-time injection” group). In both the Pre K/C-TIP1 and K/C-TIP1 groups, the injection of TIP1 dramatically decreased behavioral indicators of arthritic pain and edema in a dose-dependent manner (Fig. 6n, o). On day 5, the injection of TIP1 (10 nmol/g) reversed arthritic symptoms to almost normal levels. Moreover, the Pre K/C-TIP1 (10 nmol/g) group did not show better preventive efficacy of TIP1 when compared with group K/C-TIP1 (10 nmol/g). These results indicated that TIP1 does not affect the development of an acute arthritic condition triggered by K/C; however, its therapeutic effect is quite significant. Therefore, TIP1 may be a strong candidate for the development of new medicines for arthritis and various acute and chronic inflammatory diseases.

## Discussion

In the present study, we successfully identified a novel TLR-inhibitory peptide (TIP1) derived from the TIR domain of TIRAP. TIP1 drastically diminished the TLR4-mediated secretion of proinflammatory cytokines by specifically binding to the TIR domain of TLR4 and by blocking the transmission of downstream signals that activate the pathway. Altogether, our data support the protective effects of TIP1 against sepsis, including TIP1’s ability to relieve kidney and liver failure commonly observed in systemic inflammatory states and its ability to alleviate the symptoms of RA in both the mouse CIA model and rat K/C model. These findings suggest that TIP1 is a promising candidate for the treatment of TLR4-mediated inflammatory diseases.

TIP1 blocks TLR4 pathway activation via both MyD88-dependent and -independent (TRAM- and TRIF-dependent) pathways. Furthermore, we demonstrated that TIP1 specifically penetrates the cell membrane and binds to the TIR domain (because it remained inactive without a CPP attached to its N terminus). TIR domains in TLR families share a high degree of structural similarity, although sequence differences exist. Based on X-ray crystallographic analysis, site-directed mutagenesis, and computational predictions, a number of studies suggest that the conserved BB loop (Supplementary Fig. 4) and its adjacent regions are principal locations for TIR dimerization as well as the site of adaptor recruitment^36^. This is clearly evident from the crystal structure of the TLR10 TIR domain^37^. By means of mutagenesis data and decoy peptide approaches, alternate TIR–TIR binding surfaces involving the BB loop and helix αE have also been proposed^38^. Kagan et al. have shown that TLR4 signaling requires both TIRAP–MyD88-dependent signaling at the plasma membrane and the TRAM–TRIF-mediated pathway at the endosomal membrane after endocytosis in a sequential manner^39^. Those authors suggested the possibility of binding of adaptors TIRAP–MyD88 and TRAM–TRIF to an identical site on the TIR homodimer of TLR4. Since then, numerous studies have revealed that TIRAP’s and TRAM’s binding sites on the TIR domain of TLR4 overlap and that these adaptors compete with each other for binding and stabilizing the activated receptor^40–42^. In general, the BB loop of the TIR domain is considered important for the interaction of the TIR–TIR domain for activation of TLR signaling.

Our results indicate that the BB loop of TLR4 is an essential site for the interaction with the adaptors, and blocking this region using TIP1 disrupts both TIRAP–MyD88 and TRAM–TRIF-dependent signaling pathways^38,43^. The SPR analysis revealed that TIP1 exhibits fairly higher binding affinity for the BB loop. Thus, TIP1 targets the adaptor-binding region (i.e., BB loop) and disrupts TLR4-mediated signal transduction. The adaptors bind to the TLR-TIR domains under a membrane-anchored multimeric condition that requires a larger surface area for effective binding and stability. Therefore, based on our data, we infer that TIP1 occupies a broader surface region centering on the BB loop of TLR4 and prevents the formation of a recruitment platform for adaptor molecules. Along with TLR4, we found that TIP1 also inhibited TLR3-mediated TRAM–TRIF-dependent signaling and TLR1/2- and TLR2/6-mediated TIRAP–MyD88-dependent signaling pathways at higher concentrations. Therefore, we concluded that the peptide must have been interfering with the process of adaptor recruitment rather than receptor dimerization.

TLRs are important therapeutic candidates owing to their substantial participation in the onset of multiple diseases through stimulation of excessive secretion of proinflammatory cytokines^44^. In RA patients, the expression of TLR4 and TLR3 is significantly higher when compared with healthy individuals and is hyperresponsive to pathogen-associated molecular patterns and damage-associated molecular pattern molecules^45,46^. Eventually, the hyperactivation of the aforementioned TLRs causes the production of proinflammatory cytokines and other factors leading to the development of RA^45,47^. For instance, stimulation of RA synovial fibroblasts by RNAs from necrotic synovial-fluid cells promotes production of proinflammatory cytokines via the TLR3 signaling pathway^48^. Myeloid-related proteins 8 (Mrp8; S100A8) and 14 (Mrp14; S100A9) are expressed in neutrophils and monocytes under acute and chronic inflammatory conditions^49^ and then stimulate the production of proinflammatory cytokines after activation of NF-κB and MAPKs through the TLR4-mediated immune response^50,51^. Moreover, the concentration of high-mobility group box 1 (HMGB1) protein significantly increases in the synovial fluid of RA patients^52,53^, and it is then released from necrotic cells, triggering the production of proinflammatory cytokines via the TLR4 signaling pathway in RA^53,54^. For these reasons, the development of TLR antagonists is a perpetually active field and includes continuous improvement of these agents; at present, numerous candidates are being tested in clinical trials^3,55^. Compared with small molecules and chemicals, peptides have better efficacy, safety, selectivity, and potency, which make them attractive for future therapeutic strategies^56^. Nonetheless, the number of known antagonistic peptides that target TLRs is lower when compared with other antagonists: chemical agents and antibodies. In the present study, we found that TIP1 halts the progression of RA and ameliorates its symptoms by diminishing the TLR4-mediated production of proinflammatory cytokines. Accordingly, TIP1 can be a promising new anti-inflammatory drug candidate for the treatment of inflammatory diseases such as sepsis and autoimmune diseases, including RA, systemic lupus erythematosus, inflammatory bowel disease, multiple sclerosis, type 1 diabetes mellitus, Guillain-Barre syndrome, chronic inflammatory demyelinating polyneuropathy, or psoriasis. The peptide may also serve as a possible drug for more complex diseases such as Parkinson’s disease and Alzheimer’s disease.

## Supplementary information

Supplementary Information
