# Supplementary material for: A cell-penetrating peptide blocks Toll-like receptor-mediated downstream signaling and ameliorates autoimmune and inflammatory diseases in mice
Source: Exp Mol Med. 2019 Apr 26;51(4):50. doi: 10.1038/s12276-019-0244-0 (PMC6486608; doi:10.1038/s12276-019-0244-0)
Supplement: Supplementary file 1 — Supplementary Information [file 12276_2019_244_MOESM1_ESM.pdf]

< Supplementary information >

A cell-penetrating peptide blocks Toll-like receptor-mediated downstream signaling and ameliorates autoimmune and inflammatory diseases in mice

Hyuk-Kwon Kwon,[1,2,7] Mahesh Chandra Patra,[1] Hyeon-Jun Shin,[1] Xiangai Gui,[1] Asma Achek,[1] Suresh Panneerselvam,[1] Dong-Jin Kim,[2] Suk-Jong Song,[2] Riwon Hong,[3] Kyoung Soo Kim,[4] Yang Gyun Kim,[2] Francis Y Lee,[5] Dae-Hyun Hahm,[6] Sang Ho Lee,[2] Sangdun Choi[1*]

[1]Department of Molecular Science and Technology, Ajou University, Suwon 16499, Korea

[2]Division of Nephrology, Department of Internal Medicine, Kyung Hee University Hospital at Gangdong, Seoul 05278, Korea

[3]Department of Science in Korean Medicine, College of Korean Medicine, Kyung Hee University, Seoul 02447, Korea

[4]East-West Bone & Joint Research Institute, Kyung Hee University Hospital at Gangdong, Seoul 05278, Korea

[5]Department of Orthopaedics and Rehabilitation, Yale School of Medicine, New Haven, CT 06510, USA

[6]Department of Physiology, School of Medicine, Kyung Hee University, Seoul 02447, Korea

[7]Present address: Department of Orthopaedics and Rehabilitation, Yale School of Medicine, New Haven, CT 06510, USA

*Corresponding author: sangdunchoi@ajou.ac.kr; sangdunchoi@gmail.com
Phone: +82-31-219-2600; Fax: +82-31-219-1615

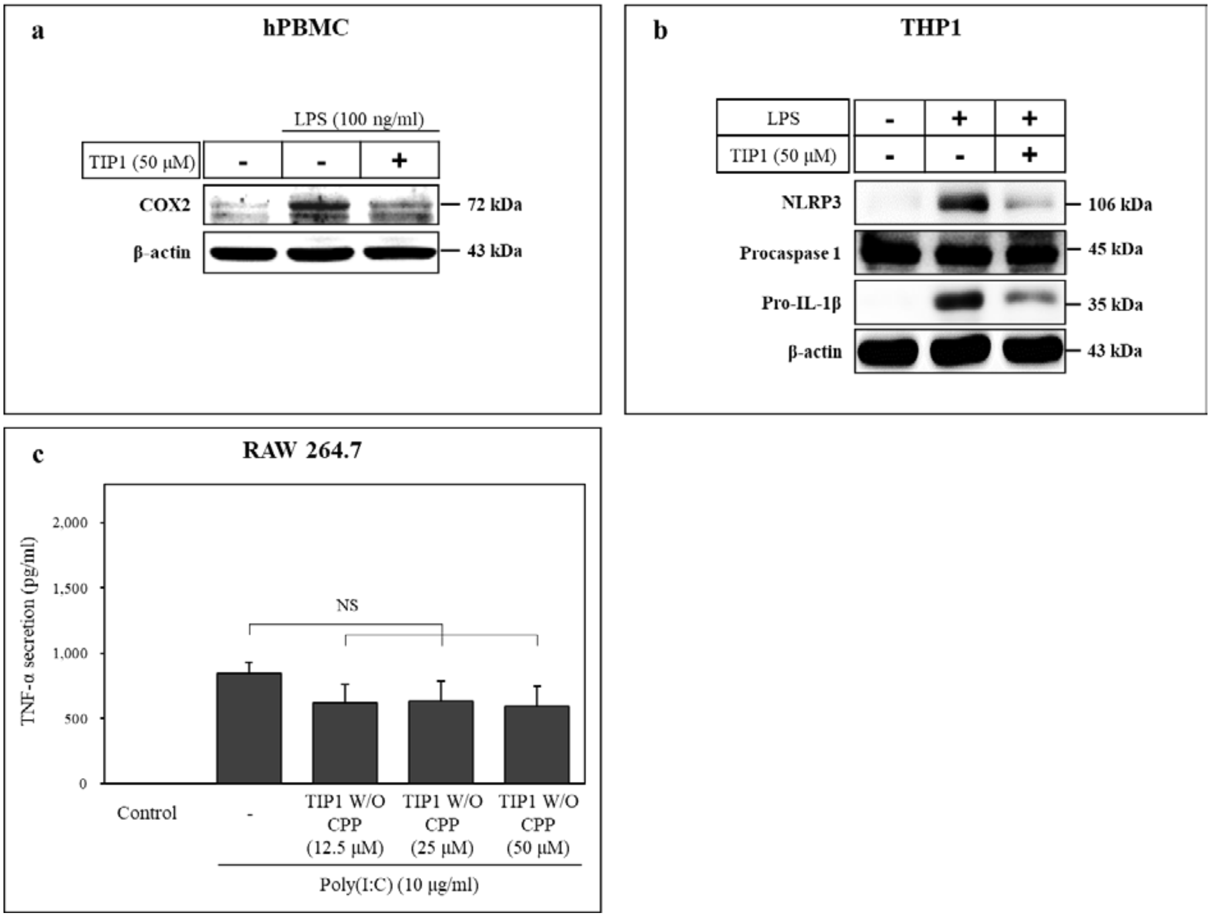

Supplementary Fig. 1 Antagonistic effects of TIP1 on TLR signaling. **(a)** hPBMCs were treated with TIP1 for 1 h followed by the treatment with LPS for 24 h. The expression of COX2 was measured by western blotting. β-Actin was used as a loading control. **(b)** THP1 cells were treated with TIP1 for 1 h before the treatment of cells with LPS for 4 h. The expression levels of NLRP3, procaspase 1 and pro-IL-1β were measured by western blotting. β-actin served as a loading control. **(c)** The secretion levels of TNF-α in RAW 264.7 cells were measured. The cells were treated with different concentrations of TIP1 nonconjugated with CPP: TIP1 without (W/O) CPP for 1 h followed by the treatment with Poly(I:C) for 24 h. Cytokine secretion was measured by an ELISA. The data shown represent at least three independent experiments ($n \geq 3$) and bars represent means ± SEM.

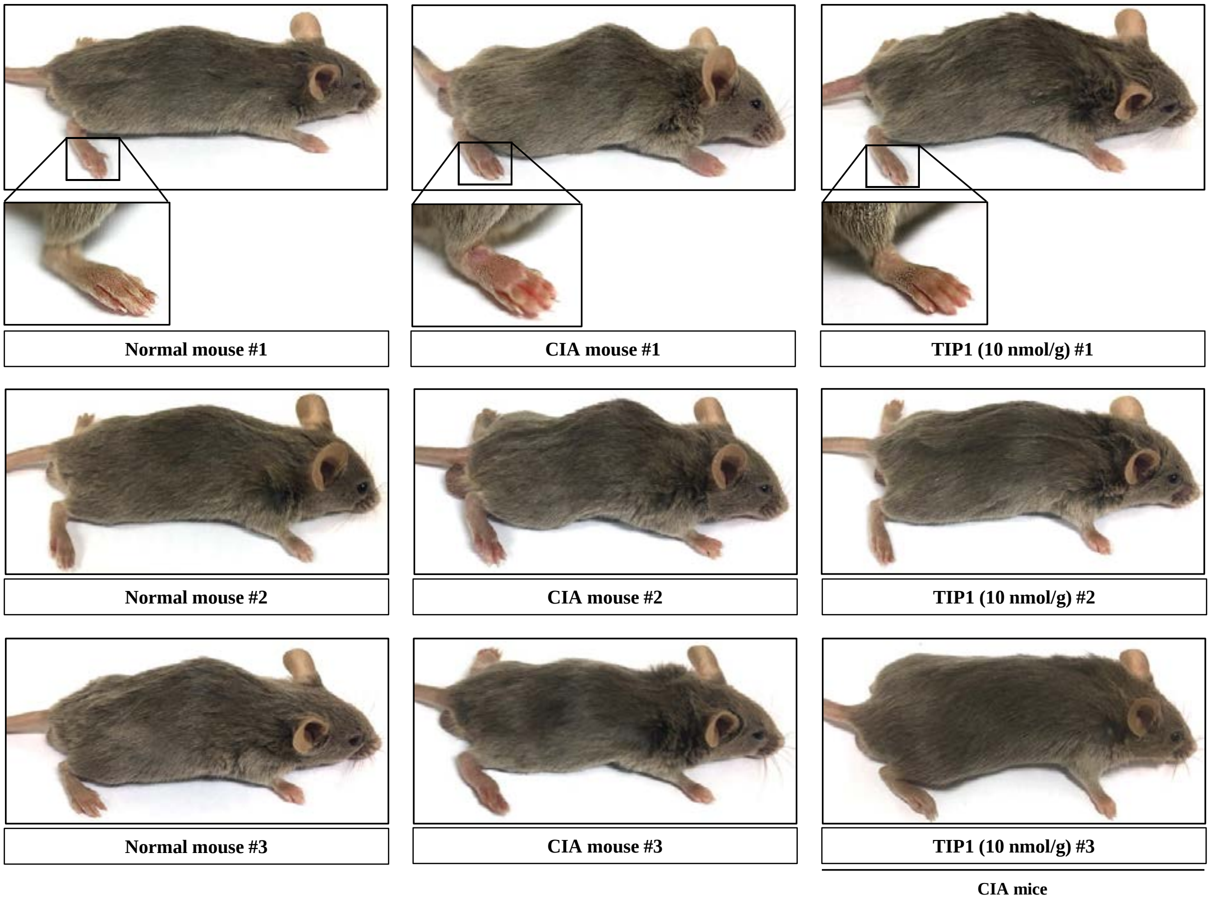

Supplementary Fig. 2 The inhibitory effects of TIP1 on the development of rheumatoid arthritis. On day 45, representative photographs show the overall shapes of paws and magnified features of right hind paws from mice in groups normal, CIA, and CIA with TIP1 (10 nmol/g) (related to Fig. 6b in the main text).

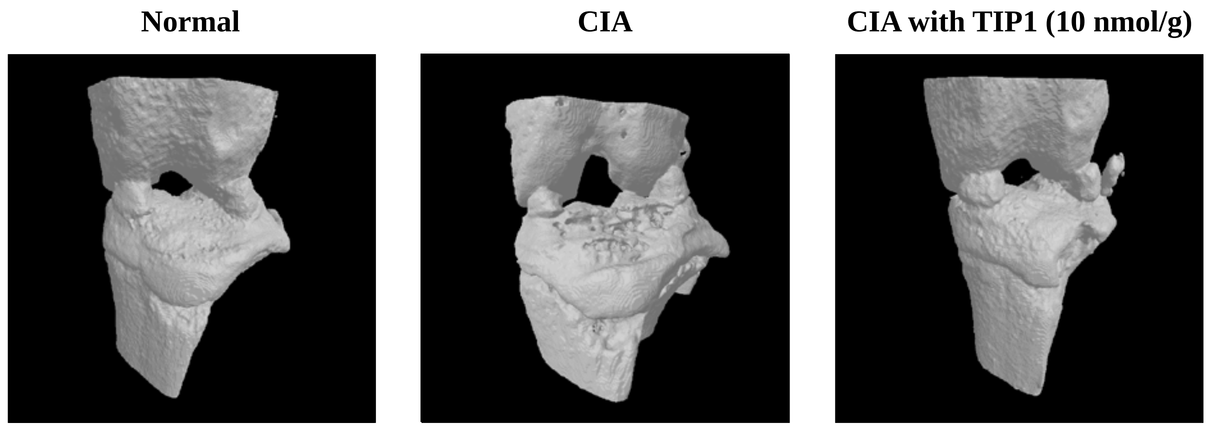

Supplementary Fig. 3 The effects of TIP1 on the disruption of knee joints. Representative three-dimensional (3D) images of knee joints from mouse groups normal, CIA, and CIA with TIP1 (10 nmol/g) were taken by Micro-CT (related to Fig. 6g in the main text).

```
                        BB loop
hTLR5|661-858       QNALLKHLDTQYSDQNRFNLCFEERDFVPGENRIANIQD-AIWNSRKIVCLVSRHFLRDG    106
mTLR5|663-859       QNALLKHLDAHYSSRNRLRLCFEERDFIPGENHISNIQA-AVWGSRKTVCLVSRHFLKDG    105
hTLR3|726-904       WEHFSSMEKEDQ----SLKFCLEERDFEAGVFELEAIVN-SIKRSRKIIFVITHHLLKDP    100
mTLR3|727-905       WEHFSPMEEQDQ----SLKFCLEERDFEAGVLGLEAIVN-SIKRSRKIIFVITHHLLKDP    100
hTLR4|653-839       RNELVKNLEEGVP---PFQLCLHYRDFIPGVAIAANIIHEGFHKSRKVIVVVSQHFIQSR    93
mTLR4|660-835       RNELVKNLEEGVP---RFHLCLHYRDFIPGVAIAANIIQEGFHKSRKVIVVVSRHFIQSR    84
hTLR2|610-784       ENLMVQELENFNP---PFKLCLHKRDFIPGKWIIDNIID-SIEKSHKTVFVLSENFVKSE    102
mTLR2|609-784       ENLMVQQLENSDP---PFKLCLHKRDFVPGKWIIDNIID-SIEKSHKTVFVLSENFVRSE    103
hTLR10|598-811      KNELIPNLEKEDG---SILICLYESYFDPGKSISENIVS-FIEKSYKSIFVLSPNFVQNE    107
hTLR1|602-786       KNELLPNLEKEG-----MQICLHERNFVPGKSIVENIIT-CIEKSYKSIFVLSPNFVQSE    104
hTLR6|608-796       KSELVPYLEKED-----IQICLHERNFVPGKSIVENIIN-CIEKSYKSIFVLSPNFVQSE    103
mTLR1|604-795       KNELLPNLEKDD-----IQICLHERNFVPGKSIVENIIN-FIEKSYKSIFVLSPHFIQSE    105
mTLR6|606-795       KNELLPNLEKDD-----IRVCLHERNFVPGKSIVENIIN-FIEKSYKAIFVLSPHFIQSE    105
hTLR9|840-1032      YNELRGQLEECRG-RWALRLCLEERDWLPGKTLFENLWA-SVYGSRKTLFVLAHTDRVSG    106
mTLR9|840-1032      YNELRVRLEERRG-RRALRLCLEDRDWLPGQTLFENLWA-SIYGSRKTLFVLAHTDRVSG    106
hTLR8|849-1041      INELRYHLEESRD-K-NVLLCLEERDWDPGLAIIDNLMQ-SINQSKKTVFVLTKKYAKSW    106
mTLR8|840-1032      INELRYHLEESED-K-SVLLCLEERDWDPGLPIIDNLMQ-SINQSKKTIFVLTKKYAKSW    106
hTLR7|861-1049      LAELVAKLEDPRE-K-HFNLCLEERDWLPGQPVLENLSQ-SIQLSKKTVFVMTDKYAKTE    105
mTLR7|859-1050      LQELVAKLEDPRE-K-HFNLCLEERDWLPGQPVLENLSQ-SIQLSKKTVFVMTQKYAKTE    108
mTLR13|805-991      YKELVPALEQGSQ--TTFKLCLHQRDFEPGIDIFENIQN-AINTSRKTLCVVSNHYLHSE    101
mTLR11|743-926      LEELVPVLEKAPPEGEGLRLCLPARDFGIGNDRMESMIA-SMGKSRATLCVLTGQALASP    105
mTLR12|731-906      IEELLPALEGFLPAGLGLRLCLPERDFEPGKDVVDNVVD-SMLSSRTTLCVLSGQALCNP    104
                       :    .        . .*:    :  *      :    .  *   : :::
```

hTLR5	C	F	E	E	R	D	F	V	P	G	E	N	R	I	A	N
mTLR5	C	F	E	E	R	D	F	I	P	G	E	N	H	I	S	N
hTLR3	C	L	E	E	R	D	F	E	A	G	V	F	E	L	E	A
mTLR3	C	L	E	E	R	D	F	E	A	G	V	L	G	L	E	A
hTLR4	C	L	H	Y	R	D	F	I	P	G	V	A	I	A	A	N
mTLR4	C	L	H	Y	R	D	F	I	P	G	V	A	I	A	A	N
hTLR2	C	L	H	K	R	D	F	I	P	G	K	W	I	I	D	N
mTLR2	C	L	H	K	R	D	F	V	P	G	K	W	I	I	D	N
hTLR10	C	L	Y	E	S	Y	F	D	P	G	K	S	I	S	E	N
hTLR1	C	L	H	E	R	N	F	V	P	G	K	S	I	V	E	N
hTLR6	C	L	H	E	R	N	F	V	P	G	K	S	I	V	E	N
mTLR1	C	L	H	E	R	N	F	V	P	G	K	S	I	V	E	N
mTLR6	C	L	H	E	R	N	F	V	P	G	K	S	I	V	E	N
hTLR9	C	L	E	E	R	D	W	L	P	G	K	T	L	F	E	N
mTLR9	C	L	E	D	R	D	W	L	P	G	Q	T	L	F	E	N
hTLR8	C	L	E	E	R	D	W	D	P	G	L	A	I	I	D	N
mTLR8	C	L	E	E	R	D	W	D	P	G	L	P	I	I	D	N
hTLR7	C	L	E	E	R	D	W	L	P	G	Q	P	V	L	E	N
mTLR7	C	L	E	E	R	D	W	L	P	G	Q	P	V	L	E	N
mTLR13	C	L	H	Q	R	D	F	E	P	G	I	D	I	F	E	N
mTLR11	C	L	P	A	R	D	F	G	I	G	N	D	R	M	E	S
mTLR12	C	L	P	E	R	D	F	E	P	G	K	D	V	V	D	N
							R11			R20						

TIP1

Identity = 12.5 %

Positive = 12.5 %

Supplementary Fig. 4 Sequence alignment between TIR domains of mouse and human TLRs. The region spanning upstream and downstream of the conserved BB loop sequences of all TLRs are shown for clarity. The identical residues are indicated by '*', conservative substitutions and semiconservative substitutions are marked with ':' and '.', respectively. The table below the alignment illustrates the residues of TIP1, which were predicted to interact with the BB loop residues (refer to Fig. 4b in the main text). Identical residues are colored red.

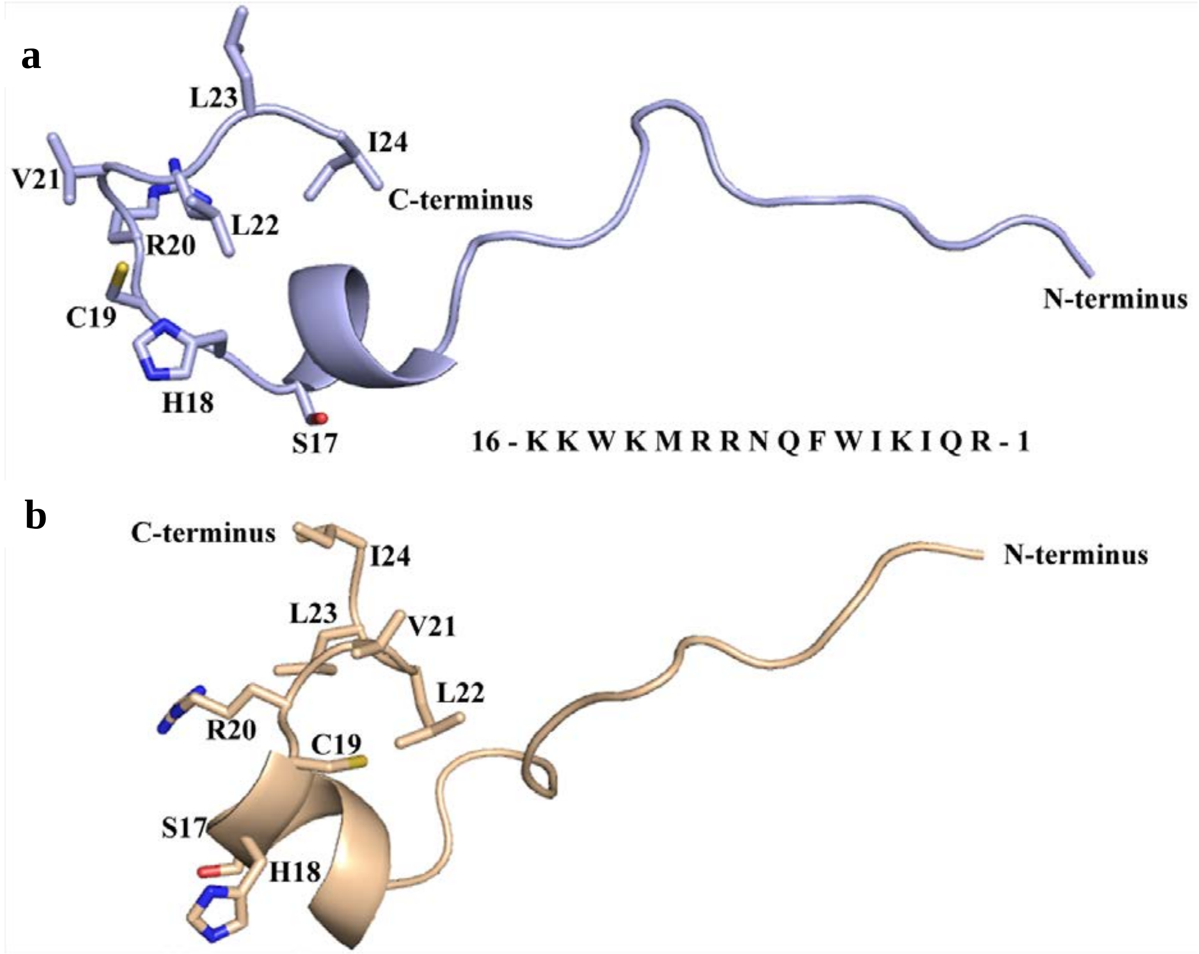

Supplementary Fig. 5 A three-dimensional model of TIP1. Representative snapshots of TIP1 before molecular dynamics (MD) simulation **(a)** and after MD simulation **(b)** are shown. The cell-penetrating peptide residues from 1 to 16 are not labeled for clarity.

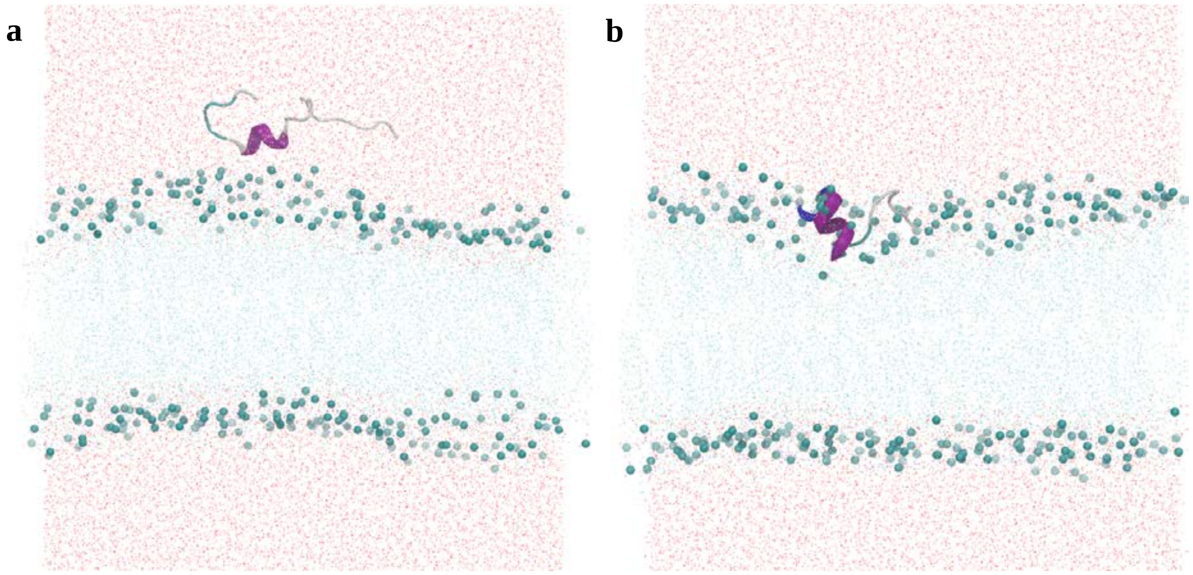

Supplementary Fig. 6 Molecular dynamics simulations of TIP1 over a dipalmitoylphosphatidylcholine bilayer. **(a)** Initial snapshot (t = 0 ns). **(b)** Final snapshot (t = 100 ns).
